# Molecular epidemiology of ESBL-producing *Escherichia coli* causing clinical and subclinical bovine mastitis: associations between multidrug resistance, virulence genes, and phylogroups

**DOI:** 10.1007/s11259-026-11214-3

**Published:** 2026-04-17

**Authors:** Elif Çokan Madenci, Farouk Hassan, Mehmet Kenan Türkyılmaz, Süheyla Türkyılmaz

**Affiliations:** 1 Institute of Health Sciences, Aydın Adnan Menderes University, Aydın, Türkiye; 2 Department of Microbiology and Immunology, Faculty of Pharmacy, Modern University for Technology and Information, Cairo, Egypt; 3 Department of Animal Science, Faculty of Veterinary Medicine, Aydın Adnan Menderes University, Aydın, Türkiye; 4 Department of Microbiology, Faculty of Veterinary Medicine, Aydın Adnan Menderes University, Aydın, Türkiye

**Keywords:** Bovine mastitis, *Escherichia coli*, Extended-spectrum beta-lactamase, Multidrug resistance, One health, Virulence factors

## Abstract

Extended-spectrum beta-lactamase (ESBL)-producing *Escherichia coli* are increasingly implicated in bovine mastitis and may serve as a reservoir of antimicrobial resistance with public health relevance. This study aimed to characterize the antimicrobial resistance, ESBL gene distribution, virulence-associated genes, integron carriage, and phylogenetic backgrounds of ESBL-producing *E.coli* isolated from bovine mastitis, and to compare these features between clinical and subclinical cases. A total of 400 bovine mastitis milk samples collected between June 2024 and May 2025 were screened for ESBL-producing bacteria, followed by phenotypic confirmation and antimicrobial susceptibility testing. Molecular characterization targeted the major ESBL genes, *bla*_CTX−M_, *bla*_TEM_, and *bla*_SHV_, class 1 and 2 integrons (*intI1*, *intI2*), 12 virulence-associated genes, and phylogenetic backgrounds determined by the Clermont method. Among 300 Gram-negative isolates, 135/300 (45.0%) *E.coli* isolates were confirmed as ESBL producers. Multidrug resistance was detected in 94/95 (98.9%) clinical isolates compared with 15/40 (37.5%) subclinical isolates, and clinical isolates showed significantly higher resistance to fluoroquinolones and aztreonam. Although *bla*_CTX−M_ predominated overall [90/95 (94.7%) in clinical mastitis and 30/40 (75.0%) in subclinical mastitis], *bla*_TEM_ and *bla*_SHV_ were also more frequent among clinical isolates [65/95 (68.4%) and 55/95 (57.9%), respectively] than among subclinical isolates [15/40 (37.5%) for both]. Class 1 integrons were common in both groups and did not differ significantly by disease presentation, whereas dual *intI1/intI2* carriage was more frequent among clinical isolates. Clinical isolates showed exclusive detection of *colV* [20/95 (21.1%) vs. 0/40], whereas subclinical isolates more frequently carried *iroN* [25/40 (62.5%) vs. 40/95 (42.1%)] and *hlyF* [30/40 (75.0%) vs. 50/95 (52.6%)]. Phylogroup B2 was more frequent among clinical isolates [45/95 (47.4%) vs. 10/40 (25.0%)], whereas phylogroup F was detected only among subclinical isolates [10/40 (25.0%) vs. 0/95]. In conclusion, ESBL-producing *E.coli* from clinical and subclinical bovine mastitis showed distinct molecular and phenotypic distributions, with clinical isolates more commonly associated with multidrug resistance, broader ESBL gene carriage, exclusive *colV* detection, and high-risk phylogenetic backgrounds. These findings support the value of integrated molecular surveillance to inform antimicrobial stewardship and One Health-oriented risk assessment in dairy production systems.

**Graphical abstract:**

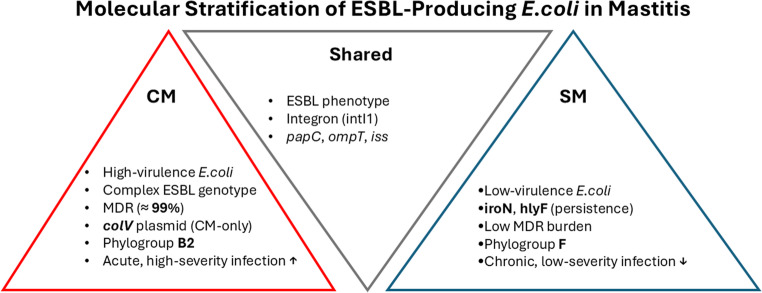

**Supplementary Information:**

The online version contains supplementary material available at 10.1007/s11259-026-11214-3.

## Introduction

Mastitis remains one of the most economically important diseases affecting the dairy industry, causing substantial treatment costs, reduced milk yield, and public health concerns due to the circulation of antimicrobial-resistant bacteria through the milk chain (Supré et al. 2021). Among environmental pathogens, *Escherichia coli* is a major cause of bovine mastitis and may induce outcomes ranging from mild, transient inflammation to severe systemic disease. The emergence of extended-spectrum beta-lactamase (ESBL)-producing *E.coli* has further complicated mastitis management by reducing the effectiveness of third-generation cephalosporins and increasing the likelihood of therapeutic failure (Bush and Bradford 2020; Garcia et al. 2024). The pathogenic potential of mastitis-associated *E.coli*, often referred to as mammary pathogenic *E.coli*, is influenced by diverse virulence determinants that may contribute to colonization, immune evasion, and persistence within the mammary gland. These include iron acquisition systems, complement resistance-associated genes, adhesins, toxins, and outer membrane proteins (Figueiredo et al. 2021). Previous studies have shown considerable variation in virulence gene repertoires among mastitis isolates, and several determinants, particularly those associated with the *colV* plasmid, may co-occur with antimicrobial resistance genes, forming adaptive genetic platforms that can enhance bacterial fitness and pathogenic potential (Goulart and Mellata 2022). A similar convergence of virulence-associated traits and antimicrobial resistance has also been reported in mastitis-associated *E.coli* from Türkiye and other regions (Güler and Gündüz 2007; Pehlivanoglu et al. 2016). Phylogenetic background is another important epidemiological feature of *E.coli*. According to the revised Clermont classification, *E.coli* populations are assigned to phylogroups that differ in ecological niche, host adaptation, and virulence potential (Clermont et al. 2019). Extraintestinal pathogenic *E.coli*-associated phylogroups, particularly B2 and D, have frequently been reported among isolates linked to more severe mastitis, higher virulence gene content, and broader antimicrobial resistance, whereas other phylogroups are more often associated with environmental persistence or subclinical infection (Nüesch-Inderbinen et al. 2019; Touchon et al. 2020). Therefore, characterization of phylogenetic structure may provide useful insight into the epidemiology of bovine mastitis and the distribution of high-risk lineages. The global spread of ESBL-producing *E.coli* in dairy herds is driven by antimicrobial selection pressure and the mobility of resistance determinants (Cardoso et al. 2022) Genomic epidemiology studies have demonstrated that high-risk ESBL lineages circulate across livestock-human interfaces and may be shared between animal and human reservoirs (Liu et al. 2023). The predominant ESBL families, especially *bla*_CTX−M_, as well as *bla*_TEM_ and *bla*_SHV_, are increasingly reported in mastitis isolates from Europe, Asia, and nearby Middle Eastern dairy systems (Ali et al. 2016; Tóth et al. 2018; Bevan et al. 2021; Obaidat et al. 2022; Penati et al. 2024). In addition, integrons facilitate the accumulation and horizontal transfer of multidrug resistance determinants (Mazel 2006; Zhang et al. 2020). Together, these trends reflect the broader antimicrobial resistance crisis in livestock production and reinforce the One Health relevance of mastitis-associated *E.coli* (Ruegg 2022;World Health Organization 2024). Despite increasing recognition of the public health importance of mastitis-associated *E.coli*, studies integrating mastitis form with antimicrobial resistance burden, ESBL gene distribution, virulence profiles, integron carriage, and phylogenetic background remain limited. In particular, comparative data distinguishing ESBL-producing *E.coli* isolated from clinical and subclinical bovine mastitis are still insufficient in many regional dairy systems. Therefore, the primary objective of this study was to characterize the distribution of antimicrobial resistance phenotypes, ESBL genes, virulence-associated genes, integron carriage, and phylogenetic groups among ESBL-producing *E.coli* isolated from bovine mastitis. The secondary objective was to compare these molecular and phenotypic features between isolates recovered from clinical and subclinical mastitis in order to identify patterns associated with disease presentation. By integrating resistance profiles, phylogenetic distribution, and virulence signatures, this study provides a regional descriptive and comparative analysis intended to support surveillance and antimicrobial stewardship within a One Health framework.

## Materials and methods

### Sample size calculation and sampling strategy

Sample size was calculated using Cochran’s formula for proportion-based sampling (Cochran 1977), based on a previously reported prevalence of 6.05% for extended-spectrum beta-lactamase (ESBL)-producing *Escherichia coli* in bovine mastitis (Tarazi et al. 2023). Because no published baseline prevalence data for ESBL-producing *E. coli* in bovine mastitis were available from Aydın Province at the time of study design, the estimate reported by Tarazi et al. (2023) was used as the closest available reference for sample size calculation. At a 95% confidence level and 5% precision, the minimum required sample size was 88. To improve the representativeness of the study and to allow comparison between clinical mastitis (CM) and subclinical mastitis (SM) isolates across phenotypic and molecular variables, the final sample size was increased to 400 milk samples. A farm-based sampling strategy was applied in Aydın Province, Türkiye. Ten dairy farms were included, and 40 milk samples were collected from each farm, yielding a total of 400 samples. The selected farms were distributed across different parts of the province to provide broad geographic coverage. Farm distribution was as follows: Söke (*n* = 1; 100 cattle), Germencik (*n* = 1; 100 cattle), Efeler (*n* = 2; 100 cattle), İncirliova (*n* = 1; 100 cattle), Nazilli (*n* = 1; 100 cattle), Kuyucak (*n* = 2; 100 cattle), Bozdoğan (*n* = 1; 100 cattle), and Çine (*n* = 1; 100 cattle). Within each farm, animals meeting the inclusion criteria were selected by random sampling.

### Sample selection and collection

Between June 2024 and May 2025, 400 milk samples were collected from dairy cows with mastitis from selected farms in Aydın Province, Türkiye, and submitted to the Microbiology Laboratory, Faculty of Veterinary Medicine, Aydın Adnan Menderes University. Cows were classified as having either clinical mastitis (CM) or subclinical mastitis (SM). To enrich the study population for treatment-refractory cases, only cows that had failed at least two previous antimicrobial treatments and had not received antimicrobial therapy within the preceding two weeks were included. Milk samples (5–10 mL) were collected aseptically after teat disinfection into sterile tubes, transported to the laboratory at 4 °C, and processed immediately upon arrival. Recovered isolates were stored at − 20 °C in accordance with National Mastitis Council guidelines (National Mastitis Council 2004).

## Classification and diagnosis of mastitis

Clinical mastitis was diagnosed by a licensed veterinarian based on udder inflammation and abnormal milk appearance, including clots, watery secretion, blood, or pus. Subclinical mastitis was identified in cows without visible clinical signs using the California Mastitis Test, and reactions were interpreted according to gel-formation scores recommended by the National Mastitis Council (National Mastitis Council 2004).

### Isolation and phenotypic identification of extended-spectrum beta-lactamase–producing *Escherichia coli*

Milk samples were pre-enriched in buffered peptone water at 37 °C for 18–24 h and cultured on blood agar, MacConkey agar, and CHROMagar™ Orientation. Colonies were purified and identified using Gram staining and standard biochemical tests according to established veterinary diagnostic protocols (Carter and Cole 1990; Quinn et al. 2011). Screening for extended-spectrum beta-lactamase production was performed on CHROMagar™ ESBL, where dark pink/red colonies were considered presumptive producers (CHROMagar 2025). Presumptive isolates were subcultured on eosin methylene blue agar and biochemically reconfirmed prior to downstream analyses. Representative colony morphologies are shown in Supplementary Figure S1.

### Phenotypic confirmation of extended-spectrum beta-lactamase production

Presumptive extended-spectrum beta-lactamase (ESBL)-producing isolates were phenotypically confirmed using the Combined Disk Test (CDT) and Double Disk Synergy Test (DDST). For CDT, a 0.5 McFarland bacterial suspension was prepared and inoculated onto Mueller-Hinton agar (MHA). Cefotaxime (CTX, 30 µg) and cefotaxime-clavulanic acid (CTX/CLA, 30/10 µg) disks were placed 30 mm apart. After incubation at 35 ± 2 °C for 18–24 h, an increase of ≥ 5 mm in the inhibition zone diameter around the CTX/CLA disk compared with CTX alone was interpreted as phenotypic confirmation of ESBL production according to CLSI criteria (CLSI 2024). All isolates positive by CDT were further evaluated by DDST. In this assay, an amoxicillin-clavulanic acid (AMC, 20/10 µg) disk was placed at the center of the plate, and ceftriaxone (CRO, 30 µg), ceftazidime (CAZ, 30 µg), cefotaxime (CTX, 30 µg), and aztreonam (ATM, 30 µg) disks were positioned 20 mm away from the central disk. Following incubation at 35 ± 2 °C for 18–24 h, enhancement of the inhibition zones toward the AMC disk was interpreted as synergism and taken as confirmation of ESBL activity (Amro et al. 2022). Isolates positive in both CDT and DDST were classified as phenotypic ESBL producers (Supplementary Figure S2).

### Antimicrobial susceptibility testing

Antimicrobial susceptibility testing was performed by the Kirby-Bauer disk diffusion method on Mueller-Hinton agar using 12 antimicrobial agents representing nine antimicrobial classes: β-lactam/β-lactamase inhibitor, second-generation cephalosporin, third-generation cephalosporins, monobactam, carbapenem, fluoroquinolones, folic acid antagonist, phenicol, and aminoglycoside (Supplementary Table S1). A 0.5 McFarland suspension of each isolate was prepared and inoculated onto the agar surface, followed by application of antibiotic disks and incubation at 35 ± 2 °C for 18–24 h. Inhibition zone diameters were measured and interpreted according to CLSI M100 zone-diameter breakpoints and interpretive criteria (CLSI 2024). *Escherichia coli* ATCC 25,922 was used as the quality control strain. Multidrug resistance (MDR) was defined as resistance to three or more antimicrobial classes according to the international consensus criteria of Magiorakos et al. (2012).

#### DNA extraction and polymerase chain reaction

Genomic and plasmid DNA were extracted from confirmed Escherichia coli isolates using the DNeasy Blood & Tissue Kit and the QIAprep Spin Miniprep Kit (Qiagen,Germany), respectively, according to the manufacturers’ instructions. DNA quality and concentration were assessed spectrophotometrically, andextracts with OD260/280 values of 1.8–2.0 were stored at −20 °C until analysis (Chen et al. 2019; Sambrook and Russell 2001). Species confirmation was performed by PCR amplification of the uspA gene using the primers described by Chen and Griffiths (1998). Phylogenetic grouping was determined using the revised Clermont scheme based on chuA, yjaA, TspE4.C2, and arpA (Clermont et al. 2000, 2004, 2013), with additional trpA and arpA-based reactions for discrimination of phylogroups C and E as described by Lescat et al. (2013), and trpBA for further phylogroup assignment according to Clermont et al. (2008). Extended-spectrum β-lactamase genes were detected by conventional PCR, including blaCTX-M and blaSHV according to Bali et al. (2010), and blaTEM according to Weill et al. (2004). Class 1 and class 2 integrons were identified by amplification of intI1 and intI2 using the protocols of Bass et al. (1999) and Goldstein et al. (2001), respectively. Virulence-associated genes were screened by conventional PCR using published primer sets. Iron acquisition-associated genes included iutA (Johnson et al. 2008), iroN (Johnson et al. 2006), and iucD (Janßen et al. 2001). Serum survival- and plasmid-associated genes included iss, vat, papC, and tsh (Ewers et al. 2004), ompT (Johnson et al. 2006), and colV (Janßen et al. 2001). Toxin-associated genes included hlyA (Paton and Paton 1998) and hlyF (Morales et al. 2004). Adhesin-associated genes included papG (Johnson and Stell 2000). PCR assays were performed in a final reaction volume of 25 μL containing FIREPol Master Mix (Solis BioDyne, Estonia), 0.4 μM of each primer, and 2.5 mM MgCl₂. Monoplex PCR was used for species confirmation, ESBL genes, integrons, and virulence markers, whereas multiplex PCR was applied for phylogroup assignment except for phylogroups C and E, which were amplified separately. Primer sequences, expected amplicon sizes, annealing temperatures, extension times, and representative amplification results are presented in Supplementary Table S2 and Supplementary Figures S3–S7. 

### Statistical analysis

Associations between mastitis form (CM vs. SM) and categorical variables, including multidrug resistance, extended-spectrum beta-lactamase genes, integrons, virulence markers, and phylogenetic groups, were assessed using Pearson’s chi-square test or Fisher’s exact test, as appropriate. Effect sizes (Cramer’s V), odds ratios with 95% confidence intervals, and exact p-values are reported in Supplementary Table S9. Differences in the number of antimicrobial classes resisted per isolate were evaluated using the Mann–Whitney U test following confirmation of non-normality by the Shapiro–Wilk test. Analyses were performed using Python 3.9 and IBM SPSS Statistics v26, with significance set at *p* < 0.05.

## Results

### Bacterial culture outcomes and distribution of mastitis-associated pathogens

Bacterial growth was detected in 320 of 400 quarter-milk samples (80.0%). Gram-negative bacteria predominated, comprising 300 isolates (93.8%), whereas Gram-positive organisms accounted for 20 isolates (6.2%). No growth was observed in 80 samples (20.0%) under aerobic conditions. Subsequent extended-spectrum beta-lactamase screening and molecular analyses were therefore restricted to the 300 Gram-negative isolates. Overall culture outcomes and pathogen distribution are summarized in Supplementary Table S3.

### Extended-spectrum beta-lactamase screening on chromogenic medium

Screening on CHROMagar™ ESBL identified 240 of 300 Gram-negative isolates (80.0%) with extended-spectrum beta-lactamase–associated coloration. Of these presumptive producers, 128 (53.3%) originated from clinical mastitis samples and 112 (46.7%) from subclinical mastitis. *Escherichia coli* was the predominant species (150/240; 62.5%), followed by *Klebsiella* spp. (40; 16.7%), *Pseudomonas* spp. (30; 12.5%), and *Acinetobacter* spp. (20; 8.3%). Among *Escherichia coli* isolates, 106 (70.7%) were recovered from clinical mastitis and 44 (29.3%) from subclinical mastitis. *Escherichia coli* occurred significantly more frequently than all other extended-spectrum beta-lactamase (ESBL) producing Gram-negative species combined (χ² test, *p* < 0.0001), with all pairwise comparisons remaining significant (*p* < 0.0001). Species distribution by mastitis form is shown in Supplementary Table S4.

### Phenotypic confirmation of extended-spectrum beta-lactamase production

Presumptive ESBL– producing colonies were purified on eosin methylene blue agar and confirmed as *Escherichia coli* using standard biochemical methods (Quinn et al. 2011). Phenotypic confirmation by the combined disk test and double disk synergy test verified extended-spectrum beta-lactamase production in 135 of 150 *Escherichia coli* isolates (90.0%). Most confirmed producers were recovered from clinical mastitis cases (95/135; 70.4%), while 40 isolates (29.6%) originated from subclinical mastitis. Representative confirmation results are shown in Supplementary Figure S2.

### Antimicrobial susceptibility profiles

**Fig. 1:**
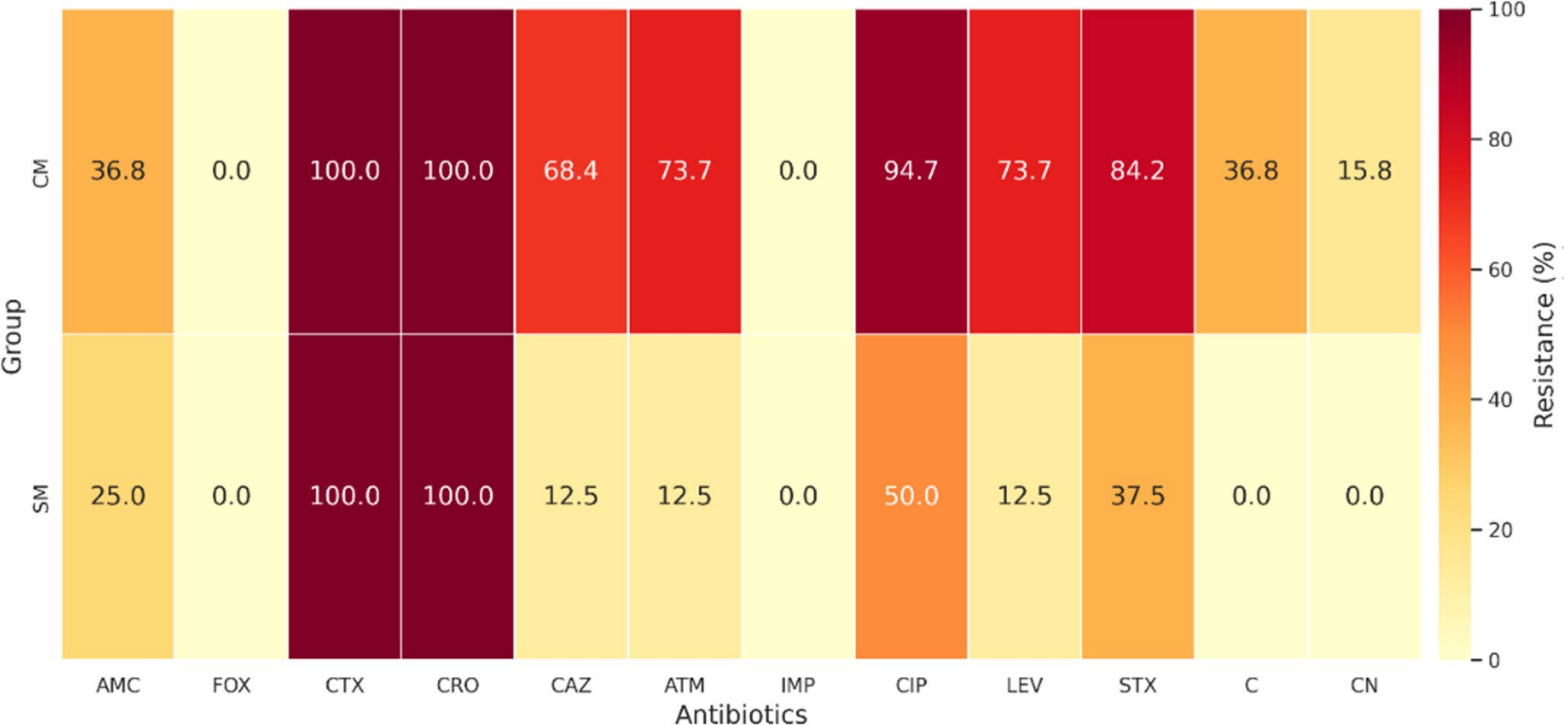
Heatmap of antimicrobial resistance profiles of ESBL-producing *E.coli* isolates from clinical (CM, *n* = 95) and subclinical (SM, *n* = 40) mastitis

Antimicrobial susceptibility testing demonstrated a clear separation in resistance severity between *Escherichia coli* isolates from clinical mastitis (CM) and subclinical mastitis (SM) (Fig. 1). Uniform resistance to cefotaxime and ceftriaxone was observed in both groups (100%), confirming widespread extended-spectrum beta-lactamase activity, while the absence of cefoxitin resistance excluded AmpC-type beta-lactamase production. CM isolates exhibited significantly higher resistance to multiple agents, including ceftazidime (68.4% vs. 12.5%; OR = 15.17; 95% CI: 5.4–42.6; *p* = 1.36 × 10⁻⁹) and aztreonam (73.7% vs. 12.5%; OR = 19.6; 95% CI: 6.9–55.6; *p* = 3.57 × 10⁻¹¹). Fluoroquinolone resistance was also markedly higher among CM isolates, including ciprofloxacin (94.7% vs. 50.0%; OR = 18.0; 95% CI: 6.0–53.7; *p* = 7.71 × 10⁻⁹) and levofloxacin (73.7% vs. 12.5%; OR = 19.6; *p* = 3.57 × 10⁻¹¹). Resistance to trimethoprim–sulfamethoxazole was likewise more frequent in CM isolates (84.2% vs. 37.5%; OR = 8.89; 95% CI: 3.8–20.7; *p* = 1.87 × 10⁻⁷). Certain resistance phenotypes were observed exclusively among CM isolates, including chloramphenicol (36.8% vs. 0%; *p* = 6.24 × 10⁻⁷) and gentamicin (15.8% vs. 0%; *p* = 0.0055). No isolate from either group exhibited resistance to imipenem. Detailed susceptibility profiles and resistance class distributions are provided in Supplementary Tables S3–S4.

### Multidrug resistance burden

**Fig. 2:**
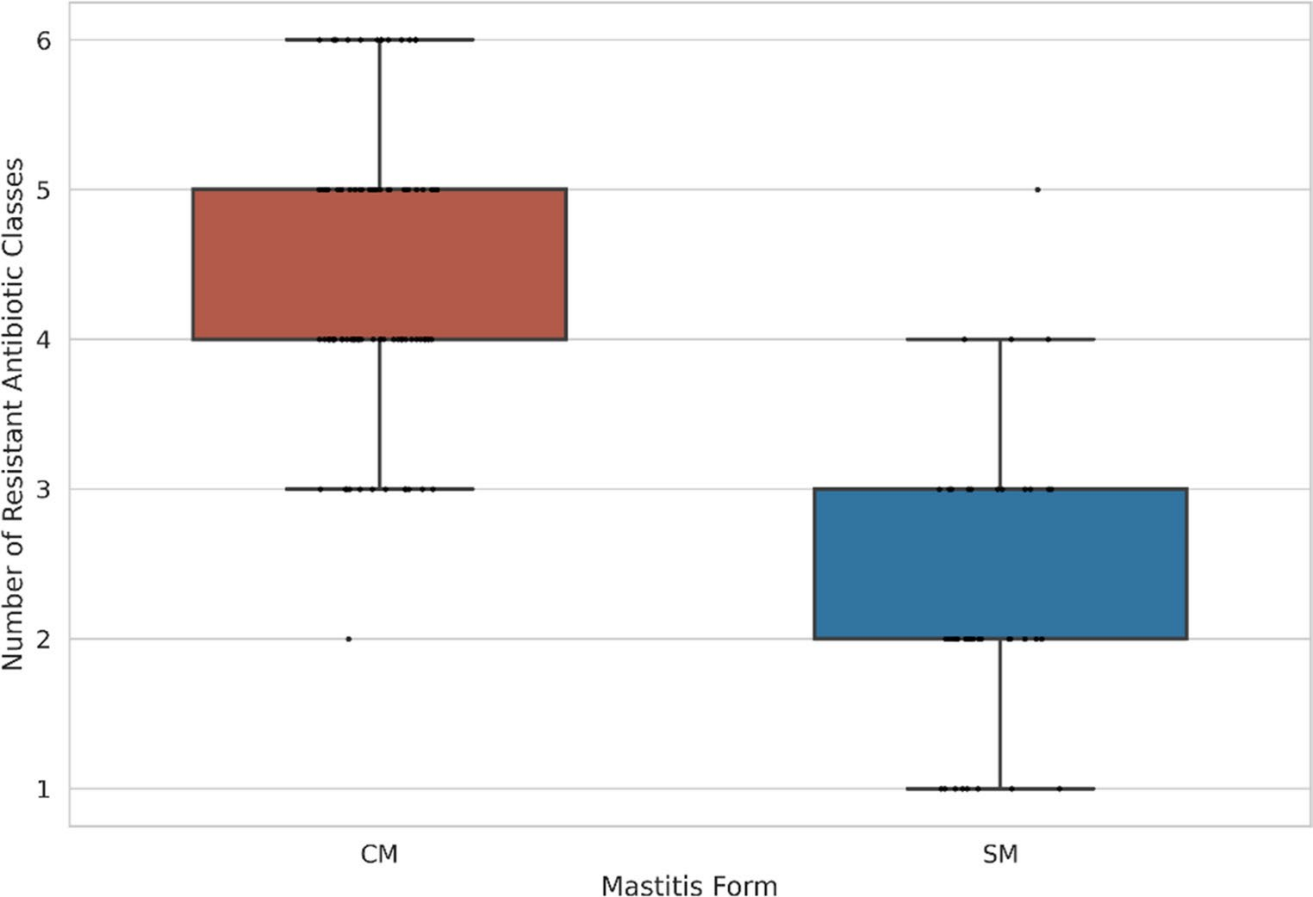
Distribution of resistant antimicrobial classes in clinical (CM) and subclinical (SM) mastitis isolates

Multidrug resistance (MDR) showed a highly asymmetric distribution between *E.coli* isolates from clinical mastitis (CM) and subclinical mastitis (SM). Nearly all CM isolates (94/95; 98.9%) met the MDR definition, compared with 15/40 (37.5%) SM isolates, demonstrating a strong association with clinical disease (Fisher’s exact test, *p* = 8.32 × 10⁻¹⁶). CM isolates were 156.7 times more likely to be multidrug resistant than SM isolates (OR = 156.7; 95% CI: 19.7–1243.8), supported by a very strong effect size (Cramer’s V = 0.691). The extent of resistance further differentiated between the two groups. CM isolates were resistant to significantly more antimicrobial classes (median 4; IQR: 4–5) than SM isolates (median 2; IQR: 2–3), as confirmed by the Mann–Whitney U test (U = 3554; *p* = 3.08 × 10⁻¹⁶). Effect size metrics indicated near-complete rank separation (*r* = 0.751; Cliff’s δ = 0.953). Resistance to six or more antimicrobial classes occurred almost exclusively among CM isolates. Detailed MDR distributions are presented in Table 1; Fig. 2, and Supplementary Table S3.

**Table 1:** Multidrug resistance (MDR) profiles of ESBL-producing *E.coli* isolates from clinical (CM) and subclinical (SM) mastitis cases

Measure	CM (*n* = 95)	SM (*n* = 40)	Statistic	*p*-value	Effect size
MDR (+)	94 (98.9%)	15 (37.5%)	OR = 156.7 (19.7–1243.8)	8.32 × 10⁻¹⁶	Cramer’s V = 0.691
MDR (–)	1 (1.1%)	25 (62.5%)	—	—	—
MDR classes	Median = 4 (IQR 4–5)	Median = 2 (IQR 2–3)	U = 3554	3.08 × 10⁻¹⁶	*r* = 0.751 δ = 0.953

### Distribution of extended-spectrum beta-lactamase genes

Molecular profiling demonstrated a significantly higher burden of extended-spectrum beta-lactamase genes among *Escherichia coli* isolates from clinical mastitis (CM) than subclinical mastitis (SM) (Table 2). The *bla*_CTX−M_ gene was the most prevalent determinant, detected in 94.7% of CM isolates versus 75.0% of SM isolates (χ² = 11.10; Fisher’s exact *p* = 0.00186; OR = 6.00; 95% CI: 1.90–18.96; Cramer’s V = 0.287). The *bla*_TEM_ gene was also significantly enriched in CM isolates (68.4% vs. 37.5%; χ² = 11.15; Fisher’s exact *p* = 0.00111; OR = 3.61; 95% CI: 1.67–7.82), as was *bla*_SHV_ (57.9% vs. 37.5%; χ² = 4.69; Fisher’s exact *p* = 0.0383; OR = 2.29; 95% CI: 1.07–4.89; Cramer’s V = 0.186). Analysis of ESBL gene combinations showed greater architectural complexity among CM isolates (Supplementary Table S9). Single-gene carriage was similar between groups, except for *bla*_*TEM*_ alone, which was more frequent in CM isolates (χ² = 4.82; *p* = 0.028). Dual-gene combinations did not differ between mastitis forms (Fisher’s exact *p* > 0.35). In contrast, triple ESBL carriage (*bla*_CTX−M_ + *bla*_TEM_ + *bla*_SHV_) was strongly associated with clinical mastitis (χ² = 15.53; Fisher’s exact *p* = 0.00004; OR = 8.97; 95% CI: 2.58–31.15; Cramer’s V = 0.339).

**Table 2:** Distribution of ESBL genes among ESBL-producing *Escherichia coli* isolates from clinical (CM) and subclinical (SM) mastitis

ESBL Gene	CM (*n* = 95)	SM (*n* = 40)	OR (95% CI)	*p*-value
*bla*_CTX−M_	90 (94.7%)	30 (75.0%)	6.00 (1.9–18.96)	0.00186
*bla*_TEM_	65 (68.4%)	15 (37.5%)	3.61 (1.67–7.82)	0.00111
*bla*_SHV_	55 (57.9%)	15 (37.5%)	2.29 (1.07–4.89)	0.0383

### Integron profiles

Screening for integron-associated integrase genes showed that class 1 integrons (*intI1*) were common among *Escherichia coli* isolates. However, *intI1* prevalence did not differ significantly between clinical mastitis (CM) and subclinical mastitis (SM) isolates (χ² = 1.776; Fisher’s exact *p* = 0.231; Cramer’s V = 0.115). Class 2 integrons (*intI2*) were not detected as single elements in any isolate. A small subset of isolates carried both *intI1* and *intI2*, and this dual-integron profile was significantly more frequent among CM isolates (χ² = 4.547; Fisher’s exact *p* = 0.0332; Cramer’s V = 0.184). The absence of integrons did not differ between CM and SM isolates (χ² = 0.025; *p* = 0.873). Detailed distributions are provided in Supplementary Tables S6 and S9.

### Virulence gene distribution

**Fig. 3:**
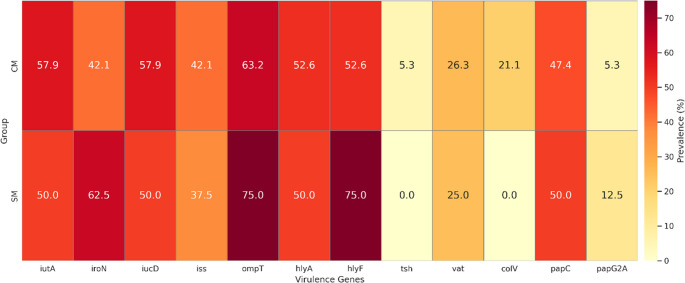
Virulence gene prevalence among ESBL-producing *E.coli* isolates from clinical (CM) and subclinical (SM) mastitis

Virulence gene profiling revealed heterogeneous distributions between Escherichia coli isolates from clinical mastitis (CM) and subclinical mastitis (SM), with only a subset of determinants showing significant differences (Fig. 3; Supplementary Tables S7 and S9). The most discriminatory marker was *colV*, which was detected exclusively in CM isolates (21.1%, 20/95) and was absent from SM isolates (0/40) (χ² = 9.886; Fisher’s exact *p* = 0.00086; Cramer’s V = 0.271). Two additional virulence genes differed significantly between mastitis forms. The iron-acquisition gene *iroN* was more prevalent among SM isolates than CM isolates (62.5% vs. 42.1%; χ² = 4.69; Fisher’s exact *p* = 0.038; OR = 0.44; Cramer’s V = 0.186). Similarly, the plasmid-associated gene *hlyF* was enriched in SM isolates (75.0% vs. 52.6%; χ² = 5.834; Fisher’s exact *p* = 0.021; OR = 0.37; Cramer’s V = 0.208). No significant differences were observed for the remaining virulence determinants (*papC*, *ompT*, *iss*, *iutA*, *iucD*, *hlyA*, *tsh*, and *vat*) (all Fisher’s exact *p* > 0.13). Complete virulence gene distributions are provided in Supplementary Tables S7 and S9.

### Phylogenetic distribution

**Fig. 4:**
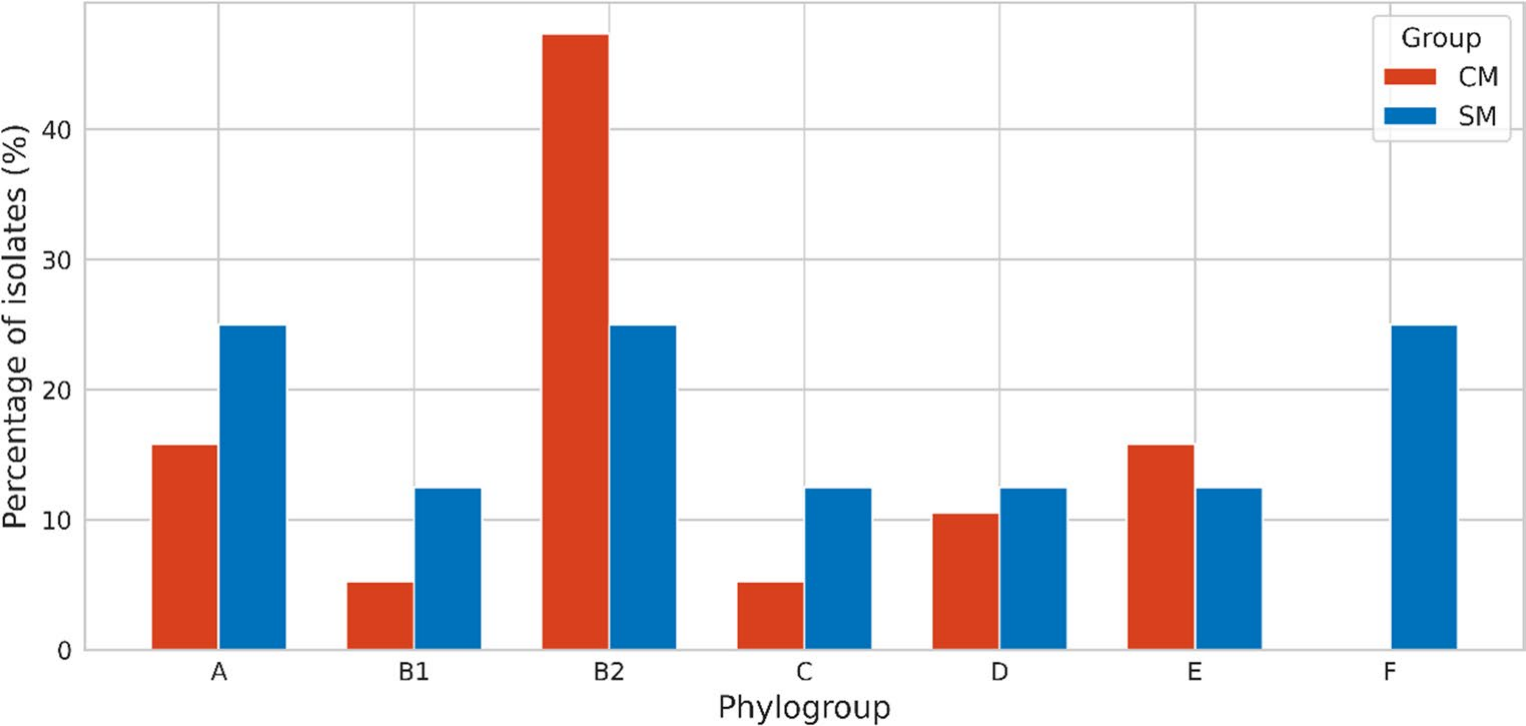
Distribution of phylogenetic groups among ESBL-producing *E.coli* from clinical (CM) and subclinical (SM) mastitis

Phylogenetic analysis demonstrated distinct lineage distributions between *Escherichia coli* isolates from clinical mastitis (CM) and subclinical mastitis (SM) (Fig. 4; Supplementary Tables S8 and S9). Phylogroup B2 was significantly more frequent among CM isolates than SM isolates (47.4% vs. 25.0%; χ² = 4.26; Fisher’s exact *p* = 0.044; OR = 2.82; 95% CI: 1.06–7.04). In contrast, phylogroup F was detected exclusively among SM isolates (25.0% vs. 0%; χ² = 8.99; Fisher’s exact *p* = 0.0009; Cramer’s V = 0.281). No significant differences were observed for phylogroups A, B1, C, D, or E between CM and SM isolates (all Fisher’s exact *p* > 0.18). Full phylogroup distributions are provided in Supplementary Tables S8 and S9.

### Integrated analysis of resistance, virulence, and phylogenetic background

Integrated analysis of antimicrobial resistance, virulence determinants, and phylogenetic background revealed clear molecular stratification between *Escherichia coli* isolates from clinical mastitis (CM) and subclinical mastitis (SM). CM isolates were defined by a high multidrug-resistance burden, elevated frequencies of extended-spectrum beta-lactamase genes, and enrichment of complex beta-lactamase architectures, including triple ESBL profiles. These resistance features co-occurred with overrepresentation of phylogroup B2 and exclusive carriage of the *colV* virulence plasmid. In contrast, SM isolates clustered predominantly within phylogroup F, displayed substantially lower multidrug resistance rates, and showed higher prevalence of persistence-associated virulence markers such as *iroN* and *hlyF*, while lacking high-impact determinants including *colV*.

## Discussion

This study provides a molecular and phenotypic comparison of ESBL-producing *Escherichia coli* associated with clinical mastitis (CM) and subclinical mastitis (SM) in dairy cattle. The high frequency of phenotypically confirmed ESBL-producing isolates, together with complete resistance to cefotaxime and ceftriaxone and preserved susceptibility to cefoxitin, indicates that ESBL-mediated β-lactam resistance was the dominant resistance pattern in the present collection, whereas AmpC-associated resistance was not supported phenotypically (Bush and Bradford 2020; CLSI 2024). This profile is consistent with reports showing the increasing establishment of ESBL-producing *E.coli* in dairy production systems (Goulart and Mellata 2022; Penati et al. 2024). In addition, CHROMagar™ ESBL yielded approximately 10% more presumptive positive isolates than were confirmed by CDT/DDST, highlighting the high screening sensitivity but lower specificity of chromogenic media and reinforcing the need for confirmatory phenotypic testing in epidemiological studies (Amro et al. 2022). A clear difference in antimicrobial resistance distribution was observed between CM and SM isolates. Clinical isolates showed significantly higher resistance to ceftazidime, aztreonam, fluoroquinolones, and trimethoprim-sulfamethoxazole, together with an almost universal multidrug-resistant phenotype. This finding agrees with previous studies in which MDR phenotypes were more frequently detected among isolates recovered from more severe mastitis presentations (Ali et al. 2016; My et al. 2023). By contrast, SM isolates showed narrower resistance profiles. Because the present study was cross-sectional, these differences should be interpreted as associations with disease presentation rather than evidence that resistance burden directly determines mastitis severity. The genotypic findings supported the same comparative pattern. Clinical isolates showed significantly higher frequencies of *bla*_CTX−M_, *bla*_TEM_, and *bla*_SHV_, and co-carriage of these genes was more common in the CM group. These multilocus ESBL profiles may reflect plasmid-associated high-risk lineages with broader resistance potential and increased opportunity for horizontal gene transfer (Bevan et al. 2021; Freitag et al. 2017; Garcia et al. 2024; Liu et al. 2023). In contrast, class 1 integrons were widely distributed in both CM and SM isolates and did not significantly distinguish disease presentation, suggesting that integron carriage may represent a broader background component of the bovine resistome rather than a specific marker of clinical form in this dataset (Mazel 2006; Zhang et al. 2020). This observation is also consistent with regional findings showing widespread integron circulation among resistant bovine isolates (Dolgun et al. 2023). Virulence profiling revealed a parallel distributional difference between the two mastitis forms. The *colV* plasmid was detected only among CM isolates, consistent with previous reports linking *colV*-associated lineages with extraintestinal virulence, immune evasion, and enhanced fitness in host-associated environments (Johnson et al. 2008; Johnson and Stell 2000). In contrast, SM isolates more frequently carried determinants such as *iroN* and *hlyF*, which have been associated with iron acquisition, persistence, and long-term colonization in some *E.coli* backgrounds (Figueiredo et al. 2021; Nüesch-Inderbinen et al. 2019). These findings suggest that CM and SM isolates differed in virulence-associated gene composition; however, given the cross-sectional design, these patterns should be interpreted as correlations rather than causal determinants of mastitis severity. Phylogenetic analysis further supported the distinction between the two groups. Clinical isolates were predominantly assigned to phylogroup B2, whereas SM isolates were more frequently associated with phylogroup F. This pattern is biologically plausible because B2 is commonly linked to extraintestinal pathogenic *E.coli* and higher virulence potential, whereas other phylogroups may be more often associated with persistence or lower pathogenicity in host and environmental settings (Clermont et al. 2019; Touchon et al. 2020). Similar lineage-related differences have been described in previous mastitis studies, although the relative frequencies of specific phylogroups vary across reports. Such discrepancies may reflect differences in geography, herd structure, sampling design, case definition, and laboratory methods rather than true biological contradiction (Tarazi et al. 2023; Zuo et al. 2025). Taken together, the results indicate that ESBL-producing *E.coli* from CM and SM differed in their distributions of multidrug resistance, ESBL gene content, virulence-associated genes, and phylogenetic background. Clinical mastitis isolates were more frequently associated with MDR, broader ESBL gene carriage, exclusive *colV* detection, and high-risk phylogenetic groups, whereas subclinical isolates were more often linked to narrower resistance profiles and persistence-associated gene patterns. From a One Health perspective, the detection of ESBL-producing and ExPEC-associated *E.coli* in dairy cattle is important because such lineages may contribute to antimicrobial resistance circulation across animal, environmental, and human interfaces (Garcia et al. 2024; WHO 2024). Overall, these findings support the presence of distinct molecular-epidemiological profiles associated with different clinical presentations of bovine mastitis.

## Conclusion

This study showed that clinical and subclinical bovine mastitis caused by ESBL-producing *Escherichia coli* differed in their distributions of antimicrobial resistance, ESBL gene content, virulence-associated profiles, and phylogenetic background. Clinical mastitis isolates were more frequently associated with multidrug resistance, broader ESBL gene carriage, *colV*-associated traits, and ExPEC-related phylogroups, whereas subclinical isolates were more commonly linked to narrower resistance profiles and persistence-associated characteristics. These findings support the presence of distinct molecular-epidemiological profiles associated with different clinical presentations of bovine mastitis. Overall, the results highlight the value of integrated bacterial profiling for surveillance and support targeted antimicrobial stewardship and One Health-oriented monitoring in dairy production systems.

### Limitations and future perspectives

This study was limited by sampling from selected farms within a single geographic region and by enrichment for treatment-refractory mastitis cases, which may restrict generalizability. Although the total sample size exceeded the minimum calculated requirement, the subgroup distribution may still have limited statistical power for some comparisons. In addition, the cross-sectional design does not allow causal inference, and the absence of whole-genome sequencing prevented detailed analysis of clonal relatedness, plasmid structure, and transmission pathways. Future studies should include multicenter or longitudinal sampling and whole-genome sequencing to better define dissemination patterns, resistance-virulence linkage, and the genetic relationships between clinical and subclinical mastitis isolates.

## Supplementary Information

Below is the link to the electronic supplementary material.

Supplementary Material 1

## Data Availability

All data generated or analyzed during this study are included in this published article and its supplementary information files. Additional raw datasets (PCR gel images, phenotypic test outputs, and antimicrobial susceptibility measurements) are available from the corresponding author on reasonable request.
