# Supplementary material for: Molecular epidemiology of ESBL-producing Escherichia coli causing clinical and subclinical bovine mastitis: associations between multidrug resistance, virulence genes, and phylogroups
Source: Vet Res Commun. 2026 Apr 17;50(4):274. doi: 10.1007/s11259-026-11214-3 (PMC13090260; doi:10.1007/s11259-026-11214-3)
Supplement: Supplementary file 1 — Supplementary Material 1 [file 11259_2026_11214_MOESM1_ESM.docx]

**Supplementary Table S1.** Antimicrobial agents used for susceptibility testing and corresponding CLSI 2024 zone-diameter breakpoints.

| **Antimicrobial family** | **Antibiotic name (Abbreviation, µg)** | **Zone diameter (mm)** | |
| --- | --- | --- | --- |
|  |  | **≥S** | **≤R** |
| β-lactam | Amoxicillin-clavulanic acid (AMC, 20/10) | 18 | 13 |
| Cephalosporin (Second Generation) | Cefoxitin (FOX, 30) | 18 | 14 |
| Cephalosporin (Third Generation) | Cefotaxime (CTX, 30) | 26 | 22 |
| Cephalosporin (Third Generation) | Ceftriaxone (CRO, 30) | 23 | 19 |
| Cephalosporin (Third Generation) | Ceftazidime (CAZ, 30) | 21 | 17 |
| Monobactam | Aztreonam (ATM, 30) | 21 | 17 |
| Carbapenem | Imipenem (IMP, 10) | 23 | 19 |
| Fluoroquinolone | Ciprofloxacin (CIP, 5) | 26 | 21 |
| Fluoroquinolone | Levofloxacin (LEV, 5) | 21 | 16 |
| Folic acid antagonist | Sulfamethoxazole/Trimethoprim (STX, 25) | 16 | 10 |
| Phenicol | Chloramphenicol (C, 30) | 18 | 12 |
| Aminoglycoside | Gentamicin (CN, 10) | 18 | 14 |

**Supplementary Table S2.** Primer sequences, amplicon sizes, and PCR conditions used for molecular characterization of Escherichia coli isolates.

| **Gene** | **Sequence (5’ to 3’)** | **Amplicon (bp)** | **Ta (^o^C), Time(sn)** | **Elongation**  **(72 ^o^C)** | **Reference(s)** |
| --- | --- | --- | --- | --- | --- |
| *usp*A | CCGATACGCTGCCAATCAG  ACGCAGACCGTAGGCCAGAT | 884 | 59,  30 | 60 s | Chen and Griffiths (1998) |
| *chuA* | ATGGTACCGGACGAACCAAC  TGCCGCCAGTACCAAAGACA | 288 | 57.5,  30 | 30 s | Clermont et al. (2000, 2013) |
| *yja*A | CAAACGTGAAGTGTCAGGAG  AATGCGTTCCTCAACCTGTG | 211 | 55.4,  30 | 15 sn | Clermont et al. (2013) |
| *TspE*4.C2 | CACTATTCGTAAGGTCATCC  AGTTTATCGCTGCGGGTCGC | 152 | 56.5,  30 | 15 sn | Clermont et al. (2013) |
| *arp*A | AACGCTATTCGCCAGCTTGC  TCTCCCCATACCGTACGCTA | 400 | 57.5,  30 | 30 s | Clermont et al. (2013, 2004) |
| *arp*A | GATTCCATCTTGTCAAAATATGCC  GAAAAGAAAAAGAATTCCCAAGAG | 301 | 56.3,  30 | 30 s | Lescat et al. (2012) |
| *trp*A | AGTTTTATGCCCAGTGCGAG  TCTGCGCCGGTCACGCCCC | 219 | 60.3,  30 | 30 s | Lescat et al. (2012) |
| *trpB*A | CGGCGATAAAGACATCTTCAC  GCAACGCGGCCTGGCGGAAG | 489 | 61,  30 | 30 s | Clermont et al. (2008) |
| *CTXM* | CGCTGTTGTTAGGAAGTGTG  GGCTGGGTGAAGTAAGTGAC | 569 | 56.5,  30 | 45 s | Bali et al. (2010) |
| *SHV* | CGCCTGTGTATTATCTCCCT  CGAGTAGTCCACCAGATCCT | 293 | 56.5,  30 | 45 s | Bali et al. (2010) |
| *TEM* | ATAAAATTCTTGAAGACGAAA GACAGTTACCAATGCTTAATCA | 1080 | 50.5,  30 | 90 s | Weill et al. (2004) |
| *int1* | CCTCCCGCACGATG ATC  TCCACGCATCGTCAGGC | 280 | 54.6,  30 | 30 s | Bass et al. (1999) |
| *int2* | TTATTGCTGGGATTAGGC  ACGGCTACCCTCTGTTATC | 233 | 51,  30 | 30 s | Goldstein et al. (2001) |
| *iut*A | GGCTGGACATCATGGGAACTGG  CGTCGGGAACGGGTAGAATCG | 302 | 63,  30 | 35 s | Johnson et al. (2008) |
| *iro*N | AATCCGGCAAAGAGACGAACCGCCT  GTTCGGGCAACCCCTGCTTTGACTTT | 553 | 66,  30 | 40 s | Johnson et al. (2006) |
| *iu*cD | ACAAAAAGTTCTATCGCTTCC  CCTGATCCAGATGATGCTC | 714 | 54,  30 | 45 s | Janben et al. (2001) |
| *iss* | ATGCAGGATAATAAGATGAAA  CTATTGTGAGCAATATACA | 290 | 47,  30 | 30 s | Ewers et al. (2004) |
| *omp*T | TCATCCCGGAAGCCTCCCTCACTACTAT  TAGCGTTTGCTGCACTGGCTTCTGATAC | 496 | 68,  30 | 40 s | Johnson et al. (2006) |
| *hlyA* | GCATCATCAAGCGTACGTTCC  AATGAGCCAAGCTGGTTAAGCT | 534 | 58,  30 | 40 s | Paton and Paton (1998) |
| *hly*F | GGCCACAGTCGTTTAGGGTGCTTACC  GGCGGTTTAGGCATTCCGATACTCAG | 450 | 67,  30 | 30 s | Morales et al. (2004) |
| *vat* | TCCTGGGACATAATGGTCAG  GTGTCAGAACGGAATTGT | 981 | 50,  30 s | 60 s | Ewers et al. (2004) |
| *pap*C | TGATATCACGCAGTCAGTAGC  CCGGCCATATTCACATAA | 500 | 50,  30 | 40 s | Ewers et al. (2004) |
| papG | CTGTAATTACGGAAGTGATTTCTG  ACTATCCGGCTCCGGATAAACCAT (2A)  TCCAGAAATAGCTCATGTAACCCG (2B) | 1070  1140 | 59,  30 | 70 s | Johnson and Stell (2000) |
| *tsh* | ACTATTCTCTGCAG AAGTC  CTTCCGATGTTCTGAACT | 825 | 52,  30 | 50 s | Ewers et al. (2004) |
| *col*V | TGGTAGAATTGTGCCAGAGCAAG  GAGCTGTTTGTAGCGAAGCC | 1180 | 58,  30 | 70 s | Janben et al. (2001) |

**Supplementary Table S3.** Full antimicrobial susceptibility matrix for all ESBL-producing *Escherichia coli* isolates (n = 135).

| **IsolateID** | **CM_SM** | **AMC** | **FOX** | **CTX** | **CRO** | **CAZ** | **ATM** | **IMP** | **CIP** | **LEV** | **STX** | **C** | **CN** | **MDR_classes** |
| --- | --- | --- | --- | --- | --- | --- | --- | --- | --- | --- | --- | --- | --- | --- |
| EC001 | CM | R | S | R | R | R | S | S | R | S | R | R | R | 6 |
| EC002 | CM | S | S | R | R | R | R | S | R | R | R | S | S | 4 |
| EC003 | CM | S | S | R | R | R | R | S | R | R | R | R | S | 5 |
| EC004 | CM | S | S | R | R | S | S | S | S | R | R | R | S | 4 |
| EC005 | CM | S | S | R | R | R | R | S | R | R | R | S | S | 4 |
| EC006 | CM | R | S | R | R | R | S | S | R | R | R | S | S | 4 |
| EC007 | CM | R | S | R | R | S | R | S | R | R | R | S | S | 5 |
| EC008 | CM | S | S | R | R | R | S | S | R | R | R | R | S | 4 |
| EC009 | CM | R | S | R | R | R | R | S | R | R | R | S | R | 6 |
| EC010 | CM | S | S | R | R | S | S | S | R | R | R | S | S | 3 |
| EC011 | CM | S | S | R | R | S | S | S | R | R | R | R | S | 4 |
| EC012 | CM | S | S | R | R | R | R | S | R | S | S | S | S | 3 |
| EC013 | CM | R | S | R | R | R | R | S | R | R | R | S | S | 5 |
| EC014 | CM | S | S | R | R | S | S | S | R | R | R | R | S | 4 |
| EC015 | CM | S | S | R | R | S | R | S | R | R | R | R | S | 5 |
| EC016 | CM | S | S | R | R | R | R | S | R | S | R | S | S | 4 |
| EC017 | CM | R | S | R | R | R | R | S | R | R | S | R | S | 5 |
| EC018 | CM | S | S | R | R | S | S | S | R | R | R | S | S | 3 |
| EC019 | CM | S | S | R | R | R | S | S | R | S | R | S | R | 4 |
| EC020 | CM | S | S | R | R | S | R | S | R | R | R | R | S | 5 |
| EC021 | CM | S | S | R | R | R | S | S | R | R | R | R | S | 4 |
| EC022 | CM | S | S | R | R | R | R | S | R | S | R | S | S | 4 |
| EC023 | CM | R | S | R | R | R | S | S | R | S | R | S | S | 4 |
| EC024 | CM | S | S | R | R | S | R | S | R | R | R | S | S | 4 |
| EC025 | CM | S | S | R | R | R | S | S | R | S | R | S | R | 4 |
| EC026 | CM | S | S | R | R | R | R | S | R | R | R | S | S | 4 |
| EC027 | CM | S | S | R | R | R | R | S | S | R | R | R | R | 6 |
| EC028 | CM | R | S | R | R | R | R | S | R | R | R | R | S | 6 |
| EC029 | CM | S | S | R | R | R | R | S | R | S | R | S | S | 4 |
| EC030 | CM | S | S | R | R | S | R | S | R | R | S | S | S | 3 |
| EC031 | CM | R | S | R | R | S | S | S | R | S | S | S | S | 3 |
| EC032 | CM | S | S | R | R | S | S | S | R | R | R | S | R | 4 |
| EC033 | CM | R | S | R | R | R | R | S | R | R | R | S | S | 5 |
| EC034 | CM | R | S | R | R | S | S | S | R | R | R | R | R | 6 |
| EC035 | CM | R | S | R | R | R | R | S | R | R | R | S | S | 5 |
| EC036 | CM | S | S | R | R | S | R | S | R | R | R | S | R | 5 |
| EC037 | CM | S | S | R | R | R | R | S | R | R | S | S | S | 3 |
| EC038 | CM | R | S | R | R | R | R | S | R | R | R | S | S | 5 |
| EC039 | CM | S | S | R | R | S | R | S | R | R | R | S | S | 4 |
| EC040 | CM | S | S | R | R | R | R | S | R | R | R | S | S | 4 |
| EC041 | CM | S | S | R | R | R | S | S | R | R | R | R | S | 4 |
| EC042 | CM | S | S | R | R | R | R | S | R | S | R | S | S | 4 |
| EC043 | CM | R | S | R | R | S | S | S | R | S | S | R | S | 4 |
| EC044 | CM | S | S | R | R | R | R | S | S | R | R | R | R | 6 |
| EC045 | CM | S | S | R | R | S | S | S | R | R | R | R | S | 4 |
| EC046 | CM | R | S | R | R | R | S | S | R | S | R | S | S | 4 |
| EC047 | CM | S | S | R | R | S | R | S | R | R | S | S | S | 3 |
| EC048 | CM | R | S | R | R | R | R | S | R | R | R | S | S | 5 |
| EC049 | CM | S | S | R | R | R | R | S | R | R | R | S | S | 4 |
| EC050 | CM | S | S | R | R | R | R | S | R | R | S | S | S | 3 |
| EC051 | CM | S | S | R | R | S | R | S | S | R | R | S | S | 4 |
| EC052 | CM | R | S | R | R | R | R | S | R | R | R | S | S | 5 |
| EC053 | CM | S | S | R | R | R | R | S | R | R | R | R | S | 5 |
| EC054 | CM | S | S | R | R | R | S | S | R | R | R | S | S | 3 |
| EC055 | CM | S | S | R | R | R | R | S | R | R | R | R | S | 5 |
| EC056 | CM | R | S | R | R | R | S | S | R | R | R | S | S | 4 |
| EC057 | CM | S | S | R | R | R | R | S | R | R | R | S | S | 4 |
| EC058 | CM | R | S | R | R | S | R | S | R | S | R | R | S | 6 |
| EC059 | CM | S | S | R | R | R | S | S | R | R | S | R | S | 3 |
| EC060 | CM | S | S | R | R | S | R | S | R | R | R | S | S | 4 |
| EC061 | CM | S | S | R | R | R | R | S | R | S | R | R | R | 6 |
| EC062 | CM | R | S | R | R | S | R | S | R | S | R | S | S | 5 |
| EC063 | CM | S | S | R | R | R | R | S | R | R | R | R | S | 5 |
| EC064 | CM | R | S | R | R | R | R | S | R | R | R | S | R | 6 |
| EC065 | CM | S | S | R | R | S | R | S | R | S | R | R | S | 5 |
| EC066 | CM | S | S | R | R | R | S | S | R | R | R | S | S | 3 |
| EC067 | CM | R | S | R | R | R | R | S | R | S | S | R | S | 5 |
| EC068 | CM | S | S | R | R | S | R | S | R | R | R | S | S | 4 |
| EC069 | CM | S | S | R | R | R | R | S | R | R | R | R | S | 5 |
| EC070 | CM | S | S | R | R | R | R | S | R | S | R | R | S | 5 |
| EC071 | CM | S | S | R | R | R | R | S | R | R | R | S | S | 4 |
| EC072 | CM | S | S | R | R | R | R | S | R | R | R | R | S | 5 |
| EC073 | CM | R | S | R | R | R | R | S | R | R | R | S | S | 5 |
| EC074 | CM | R | S | R | R | S | R | S | R | S | R | R | S | 6 |
| EC075 | CM | R | S | R | R | R | R | S | R | S | R | R | S | 6 |
| EC076 | CM | R | S | R | R | R | S | S | R | R | R | S | S | 4 |
| EC077 | CM | S | S | R | R | R | S | S | R | R | S | S | S | 2 |
| EC078 | CM | S | S | R | R | R | R | S | R | R | R | S | S | 4 |
| EC079 | CM | S | S | R | R | R | R | S | R | R | R | S | R | 5 |
| EC080 | CM | R | S | R | R | R | R | S | R | R | R | S | S | 5 |
| EC081 | CM | R | S | R | R | R | R | S | R | R | R | S | R | 6 |
| EC082 | CM | R | S | R | R | S | R | S | R | R | R | S | S | 5 |
| EC083 | CM | S | S | R | R | R | R | S | R | S | R | S | R | 5 |
| EC084 | CM | S | S | R | R | R | R | S | R | R | S | S | S | 3 |
| EC085 | CM | S | S | R | R | S | R | S | R | R | R | S | S | 4 |
| EC086 | CM | S | S | R | R | R | R | S | R | R | R | R | S | 5 |
| EC087 | CM | R | S | R | R | S | R | S | R | S | R | S | S | 5 |
| EC088 | CM | S | S | R | R | R | R | S | R | R | S | R | S | 4 |
| EC089 | CM | R | S | R | R | R | R | S | S | S | R | S | S | 4 |
| EC090 | CM | S | S | R | R | S | R | S | R | R | R | R | S | 5 |
| EC091 | CM | R | S | R | R | R | R | S | R | R | S | R | S | 5 |
| EC092 | CM | R | S | R | R | R | R | S | R | S | R | S | S | 5 |
| EC093 | CM | S | S | R | R | R | R | S | R | R | R | S | S | 4 |
| EC094 | CM | R | S | R | R | R | R | S | R | S | R | R | S | 6 |
| EC095 | CM | R | S | R | R | S | R | S | R | R | S | S | R | 5 |
| EC096 | SM | S | S | R | R | S | S | S | S | S | S | S | S | 1 |
| EC097 | SM | S | S | R | R | S | S | S | R | R | R | S | S | 3 |
| EC098 | SM | S | S | R | R | S | S | S | R | S | S | S | S | 2 |
| EC099 | SM | S | S | R | R | R | S | S | S | S | S | S | S | 1 |
| EC100 | SM | S | S | R | R | R | S | S | S | S | S | S | S | 1 |
| EC101 | SM | R | S | R | R | R | S | S | R | S | S | S | S | 3 |
| EC102 | SM | S | S | R | R | S | S | S | R | S | S | S | S | 2 |
| EC103 | SM | S | S | R | R | S | S | S | S | S | S | S | S | 1 |
| EC104 | SM | S | S | R | R | S | S | S | S | S | S | S | S | 1 |
| EC105 | SM | R | S | R | R | S | S | S | S | S | S | S | S | 2 |
| EC106 | SM | S | S | R | R | S | R | S | S | S | S | S | S | 2 |
| EC107 | SM | S | S | R | R | S | R | S | S | S | S | S | S | 2 |
| EC108 | SM | R | S | R | R | S | S | S | R | S | S | S | S | 3 |
| EC109 | SM | S | S | R | R | S | S | S | S | S | S | S | S | 1 |
| EC110 | SM | R | S | R | R | S | S | S | S | S | S | S | S | 2 |
| EC111 | SM | S | S | R | R | S | S | S | R | S | R | S | S | 3 |
| EC112 | SM | S | S | R | R | S | S | S | R | S | R | S | S | 3 |
| EC113 | SM | R | S | R | R | S | S | S | R | S | S | S | S | 3 |
| EC114 | SM | S | S | R | R | R | S | S | R | S | R | S | S | 3 |
| EC115 | SM | S | S | R | R | S | S | S | R | R | S | S | S | 2 |
| EC116 | SM | R | S | R | R | S | R | S | R | S | S | S | S | 4 |
| EC117 | SM | S | S | R | R | S | S | S | R | S | S | S | S | 2 |
| EC118 | SM | S | S | R | R | S | S | S | R | S | S | S | S | 2 |
| EC119 | SM | S | S | R | R | S | S | S | S | R | S | S | S | 2 |
| EC120 | SM | S | S | R | R | S | S | S | R | S | R | S | S | 3 |
| EC121 | SM | R | S | R | R | S | R | S | R | R | R | S | S | 5 |
| EC122 | SM | S | S | R | R | S | S | S | S | S | R | S | S | 2 |
| EC123 | SM | R | S | R | R | S | S | S | S | R | R | S | S | 4 |
| EC124 | SM | R | S | R | R | S | S | S | S | S | R | S | S | 3 |
| EC125 | SM | S | S | R | R | S | S | S | S | S | S | S | S | 1 |
| EC126 | SM | S | S | R | R | S | S | S | R | S | S | S | S | 2 |
| EC127 | SM | R | S | R | R | S | S | S | R | S | R | S | S | 4 |
| EC128 | SM | S | S | R | R | S | S | S | S | S | R | S | S | 2 |
| EC129 | SM | S | S | R | R | S | S | S | S | S | S | S | S | 1 |
| EC130 | SM | S | S | R | R | S | S | S | S | S | R | S | S | 2 |
| EC131 | SM | S | S | R | R | R | S | S | R | S | S | S | S | 2 |
| EC132 | SM | S | S | R | R | S | S | S | S | S | R | S | S | 2 |
| EC133 | SM | S | S | R | R | S | R | S | R | S | S | S | S | 3 |
| EC134 | SM | S | S | R | R | S | S | S | S | S | R | S | S | 2 |
| EC135 | SM | S | S | R | R | S | S | S | R | S | R | S | S | 3 |

**Supplementary Table S4**. Antimicrobial susceptibility profiles of ESBL-producing *Escherichia coli* isolates recovered from clinical (CM) and subclinical (SM) mastitis cases.

| **Antibiotic Class** | **Antibiotic** | **CM (n = 95)** | **SM (n = 40)** | **p (Chi²)** | **p (Fisher)** |
| --- | --- | --- | --- | --- | --- |
| **Penicillins / β-lactams** | **AMC** | 35/95 (36.8%) | 10/40 (25.0%) | 0.183 | 0.231 |
| **Cephamycin** | **FOX** | 0/95 (0%) | 0/40 (0%) | — | 1 |
| **Carbapenem** | **IMP** | 0/95 (0%) | 0/40 (0%) | — | 1 |
| **Cephalosporins (3rd gen.)** | **CTX** | 95/95 (100%) | 40/40 (100%) | — | 1 |
|  | **CRO** | 95/95 (100%) | 40/40 (100%) | — | 1 |
|  | **CAZ** | 65/95 (68.4%) | 5/40 (12.5%) | 2.9×10⁻⁹ | 1.36×10⁻⁹ |
| **Monobactams** | **ATM** | 70/95 (73.7%) | 5/40 (12.5%) | 6.46×10⁻¹¹ | 3.57×10⁻¹¹ |
| **Fluoroquinolones** | **CIP** | 90/95 (94.7%) | 20/40 (50.0%) | 9.95×10⁻¹⁰ | 7.71×10⁻⁹ |
|  | **LEV** | 70/95 (73.7%) | 5/40 (12.5%) | 6.46×10⁻¹¹ | 3.57×10⁻¹¹ |
| **Folate Pathway Inhibitors** | **STX** | 80/95 (84.2%) | 15/40 (37.5%) | 5.72×10⁻⁸ | 1.87×10⁻⁷ |
| **Chloramphenicol** | **C** | 35/95 (36.8%) | 0/40 (0%) | 8.18×10⁻⁶ | 6.24×10⁻⁷ |
| **Aminoglycosides** | **CN** | 15/95 (15.8%) | 0/40 (0%) | 0.0077 | 0.0055 |

**Supplementary Table S5.** ESBL gene combination profiles of ESBL-producing *Escherichia coli* isolates recovered from clinical (CM) and subclinical (SM) mastitis cases (n = 135).

| **IsolateID** | **CM_SM** | ***bla_CTX_M_*** | ***bla_TEM_*** | ***bla_SHV_*** | **Combination_Category** |
| --- | --- | --- | --- | --- | --- |
| EC001 | CM | 1 | 0 | 1 | blaCTX_M+blaSHV |
| EC002 | CM | 1 | 0 | 1 | blaCTX_M+blaSHV |
| EC003 | CM | 1 | 1 | 0 | blaCTX_M+blaTEM |
| EC004 | CM | 1 | 0 | 1 | blaCTX_M+blaSHV |
| EC005 | CM | 1 | 1 | 1 | blaCTX_M+blaTEM+blaSHV |
| EC006 | CM | 1 | 1 | 1 | blaCTX_M+blaTEM+blaSHV |
| EC007 | CM | 0 | 0 | 0 |  |
| EC008 | CM | 1 | 1 | 0 | blaCTX_M+blaTEM |
| EC009 | CM | 1 | 1 | 1 | blaCTX_M+blaTEM+blaSHV |
| EC010 | CM | 1 | 1 | 0 | blaCTX_M+blaTEM |
| EC011 | CM | 1 | 1 | 0 | blaCTX_M+blaTEM |
| EC012 | CM | 0 | 0 | 1 | blaSHV |
| EC013 | CM | 1 | 0 | 1 | blaCTX_M+blaSHV |
| EC014 | CM | 1 | 1 | 0 | blaCTX_M+blaTEM |
| EC015 | CM | 1 | 1 | 1 | blaCTX_M+blaTEM+blaSHV |
| EC016 | CM | 1 | 1 | 1 | blaCTX_M+blaTEM+blaSHV |
| EC017 | CM | 1 | 1 | 1 | blaCTX_M+blaTEM+blaSHV |
| EC018 | CM | 1 | 1 | 1 | blaCTX_M+blaTEM+blaSHV |
| EC019 | CM | 1 | 0 | 0 | blaCTX_M |
| EC020 | CM | 1 | 1 | 1 | blaCTX_M+blaTEM+blaSHV |
| EC021 | CM | 1 | 1 | 1 | blaCTX_M+blaTEM+blaSHV |
| EC022 | CM | 1 | 1 | 0 | blaCTX_M+blaTEM |
| EC023 | CM | 1 | 1 | 0 | blaCTX_M+blaTEM |
| EC024 | CM | 1 | 1 | 1 | blaCTX_M+blaTEM+blaSHV |
| EC025 | CM | 1 | 0 | 0 | blaCTX_M |
| EC026 | CM | 1 | 1 | 1 | blaCTX_M+blaTEM+blaSHV |
| EC027 | CM | 1 | 1 | 1 | blaCTX_M+blaTEM+blaSHV |
| EC028 | CM | 1 | 1 | 1 | blaCTX_M+blaTEM+blaSHV |
| EC029 | CM | 1 | 0 | 0 | blaCTX_M |
| EC030 | CM | 1 | 1 | 1 | blaCTX_M+blaTEM+blaSHV |
| EC031 | CM | 1 | 1 | 1 | blaCTX_M+blaTEM+blaSHV |
| EC032 | CM | 1 | 1 | 1 | blaCTX_M+blaTEM+blaSHV |
| EC033 | CM | 1 | 0 | 0 | blaCTX_M |
| EC034 | CM | 1 | 1 | 0 | blaCTX_M+blaTEM |
| EC035 | CM | 1 | 0 | 0 | blaCTX_M |
| EC036 | CM | 1 | 1 | 1 | blaCTX_M+blaTEM+blaSHV |
| EC037 | CM | 1 | 1 | 1 | blaCTX_M+blaTEM+blaSHV |
| EC038 | CM | 0 | 1 | 1 | blaTEM+blaSHV |
| EC039 | CM | 1 | 1 | 0 | blaCTX_M+blaTEM |
| EC040 | CM | 1 | 1 | 1 | blaCTX_M+blaTEM+blaSHV |
| EC041 | CM | 1 | 0 | 0 | blaCTX_M |
| EC042 | CM | 1 | 1 | 1 | blaCTX_M+blaTEM+blaSHV |
| EC043 | CM | 1 | 1 | 1 | blaCTX_M+blaTEM+blaSHV |
| EC044 | CM | 1 | 0 | 0 | blaCTX_M |
| EC045 | CM | 1 | 0 | 0 | blaCTX_M |
| EC046 | CM | 0 | 0 | 1 | blaSHV |
| EC047 | CM | 1 | 1 | 1 | blaCTX_M+blaTEM+blaSHV |
| EC048 | CM | 1 | 1 | 1 | blaCTX_M+blaTEM+blaSHV |
| EC049 | CM | 1 | 1 | 0 | blaCTX_M+blaTEM |
| EC050 | CM | 1 | 0 | 1 | blaCTX_M+blaSHV |
| EC051 | CM | 1 | 1 | 1 | blaCTX_M+blaTEM+blaSHV |
| EC052 | CM | 1 | 1 | 0 | blaCTX_M+blaTEM |
| EC053 | CM | 1 | 1 | 1 | blaCTX_M+blaTEM+blaSHV |
| EC054 | CM | 1 | 1 | 1 | blaCTX_M+blaTEM+blaSHV |
| EC055 | CM | 1 | 1 | 0 | blaCTX_M+blaTEM |
| EC056 | CM | 1 | 1 | 0 | blaCTX_M+blaTEM |
| EC057 | CM | 1 | 0 | 0 | blaCTX_M |
| EC058 | CM | 1 | 1 | 1 | blaCTX_M+blaTEM+blaSHV |
| EC059 | CM | 1 | 0 | 0 | blaCTX_M |
| EC060 | CM | 1 | 1 | 0 | blaCTX_M+blaTEM |
| EC061 | CM | 1 | 1 | 0 | blaCTX_M+blaTEM |
| EC062 | CM | 1 | 0 | 1 | blaCTX_M+blaSHV |
| EC063 | CM | 1 | 1 | 1 | blaCTX_M+blaTEM+blaSHV |
| EC064 | CM | 1 | 1 | 0 | blaCTX_M+blaTEM |
| EC065 | CM | 1 | 1 | 1 | blaCTX_M+blaTEM+blaSHV |
| EC066 | CM | 1 | 0 | 0 | blaCTX_M |
| EC067 | CM | 1 | 1 | 0 | blaCTX_M+blaTEM |
| EC068 | CM | 1 | 0 | 0 | blaCTX_M |
| EC069 | CM | 1 | 1 | 1 | blaCTX_M+blaTEM+blaSHV |
| EC070 | CM | 1 | 1 | 1 | blaCTX_M+blaTEM+blaSHV |
| EC071 | CM | 1 | 0 | 1 | blaCTX_M+blaSHV |
| EC072 | CM | 1 | 1 | 0 | blaCTX_M+blaTEM |
| EC073 | CM | 1 | 1 | 0 | blaCTX_M+blaTEM |
| EC074 | CM | 1 | 0 | 0 | blaCTX_M |
| EC075 | CM | 1 | 1 | 0 | blaCTX_M+blaTEM |
| EC076 | CM | 1 | 1 | 1 | blaCTX_M+blaTEM+blaSHV |
| EC077 | CM | 1 | 0 | 1 | blaCTX_M+blaSHV |
| EC078 | CM | 1 | 1 | 1 | blaCTX_M+blaTEM+blaSHV |
| EC079 | CM | 1 | 0 | 1 | blaCTX_M+blaSHV |
| EC080 | CM | 1 | 1 | 1 | blaCTX_M+blaTEM+blaSHV |
| EC081 | CM | 1 | 1 | 1 | blaCTX_M+blaTEM+blaSHV |
| EC082 | CM | 1 | 1 | 0 | blaCTX_M+blaTEM |
| EC083 | CM | 1 | 1 | 1 | blaCTX_M+blaTEM+blaSHV |
| EC084 | CM | 1 | 1 | 0 | blaCTX_M+blaTEM |
| EC085 | CM | 1 | 1 | 1 | blaCTX_M+blaTEM+blaSHV |
| EC086 | CM | 1 | 1 | 0 | blaCTX_M+blaTEM |
| EC087 | CM | 1 | 0 | 1 | blaCTX_M+blaSHV |
| EC088 | CM | 0 | 1 | 1 | blaTEM+blaSHV |
| EC089 | CM | 1 | 0 | 1 | blaCTX_M+blaSHV |
| EC090 | CM | 1 | 1 | 1 | blaCTX_M+blaTEM+blaSHV |
| EC091 | CM | 1 | 0 | 0 | blaCTX_M |
| EC092 | CM | 1 | 1 | 1 | blaCTX_M+blaTEM+blaSHV |
| EC093 | CM | 1 | 0 | 0 | blaCTX_M |
| EC094 | CM | 1 | 0 | 0 | blaCTX_M |
| EC095 | CM | 1 | 1 | 1 | blaCTX_M+blaTEM+blaSHV |
| EC096 | SM | 0 | 0 | 1 | blaSHV |
| EC097 | SM | 0 | 1 | 1 | blaTEM+blaSHV |
| EC098 | SM | 1 | 0 | 0 | blaCTX_M |
| EC099 | SM | 0 | 0 | 0 |  |
| EC100 | SM | 1 | 0 | 1 | blaCTX_M+blaSHV |
| EC101 | SM | 1 | 1 | 1 | blaCTX_M+blaTEM+blaSHV |
| EC102 | SM | 1 | 1 | 0 | blaCTX_M+blaTEM |
| EC103 | SM | 1 | 0 | 0 | blaCTX_M |
| EC104 | SM | 1 | 1 | 0 | blaCTX_M+blaTEM |
| EC105 | SM | 1 | 1 | 0 | blaCTX_M+blaTEM |
| EC106 | SM | 1 | 0 | 1 | blaCTX_M+blaSHV |
| EC107 | SM | 1 | 0 | 0 | blaCTX_M |
| EC108 | SM | 0 | 1 | 0 | blaTEM |
| EC109 | SM | 1 | 0 | 0 | blaCTX_M |
| EC110 | SM | 1 | 1 | 1 | blaCTX_M+blaTEM+blaSHV |
| EC111 | SM | 1 | 1 | 0 | blaCTX_M+blaTEM |
| EC112 | SM | 0 | 0 | 1 | blaSHV |
| EC113 | SM | 1 | 0 | 0 | blaCTX_M |
| EC114 | SM | 1 | 0 | 1 | blaCTX_M+blaSHV |
| EC115 | SM | 1 | 0 | 0 | blaCTX_M |
| EC116 | SM | 1 | 1 | 1 | blaCTX_M+blaTEM+blaSHV |
| EC117 | SM | 1 | 0 | 0 | blaCTX_M |
| EC118 | SM | 1 | 0 | 0 | blaCTX_M |
| EC119 | SM | 1 | 0 | 1 | blaCTX_M+blaSHV |
| EC120 | SM | 1 | 1 | 0 | blaCTX_M+blaTEM |
| EC121 | SM | 1 | 0 | 0 | blaCTX_M |
| EC122 | SM | 1 | 1 | 0 | blaCTX_M+blaTEM |
| EC123 | SM | 1 | 0 | 1 | blaCTX_M+blaSHV |
| EC124 | SM | 1 | 1 | 0 | blaCTX_M+blaTEM |
| EC125 | SM | 0 | 0 | 1 | blaSHV |
| EC126 | SM | 1 | 0 | 0 | blaCTX_M |
| EC127 | SM | 0 | 1 | 0 | blaTEM |
| EC128 | SM | 0 | 0 | 0 |  |
| EC129 | SM | 1 | 0 | 0 | blaCTX_M |
| EC130 | SM | 1 | 1 | 0 | blaCTX_M+blaTEM |
| EC131 | SM | 1 | 0 | 1 | blaCTX_M+blaSHV |
| EC132 | SM | 1 | 0 | 0 | blaCTX_M |
| EC133 | SM | 1 | 0 | 1 | blaCTX_M+blaSHV |
| EC134 | SM | 0 | 0 | 0 |  |
| EC135 | SM | 0 | 1 | 1 | blaTEM+blaSHV |

**Supplementary Table S6.** Integron profiles of ESBL-producing *Escherichia coli* isolates recovered from clinical (CM) and subclinical (SM) mastitis cases (n = 135).

| **IsolateID** | **CM_SM** | **intI1** | **intI2** | **Integron Category** |
| --- | --- | --- | --- | --- |
| EC001 | CM | 0 | 0 | none |
| EC002 | CM | 1 | 0 | int1_only |
| EC003 | CM | 0 | 0 | none |
| EC004 | CM | 1 | 0 | int1_only |
| EC005 | CM | 0 | 0 | none |
| EC006 | CM | 1 | 0 | int1_only |
| EC007 | CM | 1 | 0 | int1_only |
| EC008 | CM | 0 | 0 | none |
| EC009 | CM | 1 | 0 | int1_only |
| EC010 | CM | 1 | 0 | int1_only |
| EC011 | CM | 1 | 0 | int1_only |
| EC012 | CM | 1 | 0 | int1_only |
| EC013 | CM | 0 | 0 | none |
| EC014 | CM | 1 | 1 | int1_int2 |
| EC015 | CM | 1 | 1 | int1_int2 |
| EC016 | CM | 1 | 0 | int1_only |
| EC017 | CM | 1 | 0 | int1_only |
| EC018 | CM | 0 | 0 | none |
| EC019 | CM | 1 | 0 | int1_only |
| EC020 | CM | 1 | 0 | int1_only |
| EC021 | CM | 0 | 0 | none |
| EC022 | CM | 1 | 0 | int1_only |
| EC023 | CM | 0 | 0 | none |
| EC024 | CM | 1 | 0 | int1_only |
| EC025 | CM | 1 | 1 | int1_int2 |
| EC026 | CM | 1 | 0 | int1_only |
| EC027 | CM | 0 | 0 | none |
| EC028 | CM | 1 | 0 | int1_only |
| EC029 | CM | 1 | 0 | int1_only |
| EC030 | CM | 1 | 0 | int1_only |
| EC031 | CM | 1 | 0 | int1_only |
| EC032 | CM | 0 | 0 | none |
| EC033 | CM | 1 | 1 | int1_int2 |
| EC034 | CM | 0 | 0 | none |
| EC035 | CM | 1 | 0 | int1_only |
| EC036 | CM | 1 | 0 | int1_only |
| EC037 | CM | 1 | 0 | int1_only |
| EC038 | CM | 0 | 0 | none |
| EC039 | CM | 0 | 0 | none |
| EC040 | CM | 1 | 1 | int1_int2 |
| EC041 | CM | 1 | 0 | int1_only |
| EC042 | CM | 1 | 0 | int1_only |
| EC043 | CM | 1 | 0 | int1_only |
| EC044 | CM | 1 | 0 | int1_only |
| EC045 | CM | 1 | 0 | int1_only |
| EC046 | CM | 0 | 0 | none |
| EC047 | CM | 1 | 0 | int1_only |
| EC048 | CM | 1 | 1 | int1_int2 |
| EC049 | CM | 1 | 0 | int1_only |
| EC050 | CM | 1 | 0 | int1_only |
| EC051 | CM | 1 | 0 | int1_only |
| EC052 | CM | 1 | 0 | int1_only |
| EC053 | CM | 1 | 0 | int1_only |
| EC054 | CM | 0 | 0 | none |
| EC055 | CM | 1 | 1 | int1_int2 |
| EC056 | CM | 1 | 1 | int1_int2 |
| EC057 | CM | 1 | 0 | int1_only |
| EC058 | CM | 1 | 1 | int1_int2 |
| EC059 | CM | 1 | 0 | int1_only |
| EC060 | CM | 1 | 0 | int1_only |
| EC061 | CM | 1 | 0 | int1_only |
| EC062 | CM | 0 | 0 | none |
| EC063 | CM | 1 | 0 | int1_only |
| EC064 | CM | 0 | 0 | none |
| EC065 | CM | 1 | 1 | int1_int2 |
| EC066 | CM | 1 | 0 | int1_only |
| EC067 | CM | 1 | 0 | int1_only |
| EC068 | CM | 1 | 0 | int1_only |
| EC069 | CM | 0 | 0 | none |
| EC070 | CM | 1 | 0 | int1_only |
| EC071 | CM | 0 | 0 | none |
| EC072 | CM | 1 | 0 | int1_only |
| EC073 | CM | 0 | 0 | none |
| EC074 | CM | 1 | 0 | int1_only |
| EC075 | CM | 1 | 0 | int1_only |
| EC076 | CM | 1 | 0 | int1_only |
| EC077 | CM | 1 | 0 | int1_only |
| EC078 | CM | 0 | 0 | none |
| EC079 | CM | 1 | 0 | int1_only |
| EC080 | CM | 1 | 0 | int1_only |
| EC081 | CM | 1 | 0 | int1_only |
| EC082 | CM | 0 | 0 | none |
| EC083 | CM | 0 | 0 | none |
| EC084 | CM | 1 | 0 | int1_only |
| EC085 | CM | 1 | 0 | int1_only |
| EC086 | CM | 1 | 0 | int1_only |
| EC087 | CM | 0 | 0 | none |
| EC088 | CM | 1 | 0 | int1_only |
| EC089 | CM | 1 | 0 | int1_only |
| EC090 | CM | 1 | 0 | int1_only |
| EC091 | CM | 0 | 0 | none |
| EC092 | CM | 1 | 0 | int1_only |
| EC093 | CM | 1 | 0 | int1_only |
| EC094 | CM | 1 | 0 | int1_only |
| EC095 | CM | 1 | 0 | int1_only |
| EC096 | SM | 0 | 0 | none |
| EC097 | SM | 0 | 0 | none |
| EC098 | SM | 1 | 0 | int1_only |
| EC099 | SM | 1 | 0 | int1_only |
| EC100 | SM | 1 | 0 | int1_only |
| EC101 | SM | 1 | 0 | int1_only |
| EC102 | SM | 1 | 0 | int1_only |
| EC103 | SM | 1 | 0 | int1_only |
| EC104 | SM | 1 | 0 | int1_only |
| EC105 | SM | 0 | 0 | none |
| EC106 | SM | 1 | 0 | int1_only |
| EC107 | SM | 1 | 0 | int1_only |
| EC108 | SM | 0 | 0 | none |
| EC109 | SM | 1 | 0 | int1_only |
| EC110 | SM | 1 | 0 | int1_only |
| EC111 | SM | 1 | 0 | int1_only |
| EC112 | SM | 1 | 0 | int1_only |
| EC113 | SM | 1 | 0 | int1_only |
| EC114 | SM | 1 | 0 | int1_only |
| EC115 | SM | 1 | 0 | int1_only |
| EC116 | SM | 0 | 0 | none |
| EC117 | SM | 1 | 0 | int1_only |
| EC118 | SM | 1 | 0 | int1_only |
| EC119 | SM | 1 | 0 | int1_only |
| EC120 | SM | 1 | 0 | int1_only |
| EC121 | SM | 1 | 0 | int1_only |
| EC122 | SM | 1 | 0 | int1_only |
| EC123 | SM | 1 | 0 | int1_only |
| EC124 | SM | 0 | 0 | none |
| EC125 | SM | 1 | 0 | int1_only |
| EC126 | SM | 0 | 0 | none |
| EC127 | SM | 1 | 0 | int1_only |
| EC128 | SM | 0 | 0 | none |
| EC129 | SM | 1 | 0 | int1_only |
| EC130 | SM | 1 | 0 | int1_only |
| EC131 | SM | 0 | 0 | none |
| EC132 | SM | 1 | 0 | int1_only |
| EC133 | SM | 0 | 0 | none |
| EC134 | SM | 1 | 0 | int1_only |
| EC135 | SM | 1 | 0 | int1_only |

**Supplementary Table S7.** Virulence gene profiles of ESBL-producing *Escherichia coli* isolates recovered from clinical (CM) and subclinical (SM) mastitis cases (n = 135).

| **IsolateID** | **CM_SM** | **iutA** | **iroN** | **iucD** | **iss** | **ompT** | **hlyA** | **hlyF** | **tsh** | **vat** | **colV** | **papC** | **papG2A** |
| --- | --- | --- | --- | --- | --- | --- | --- | --- | --- | --- | --- | --- | --- |
| EC001 | CM | 1 | 1 | 0 | 1 | 1 | 0 | 1 | 0 | 0 | 0 | 0 | 0 |
| EC002 | CM | 1 | 0 | 0 | 0 | 1 | 0 | 0 | 0 | 1 | 0 | 0 | 0 |
| EC003 | CM | 0 | 1 | 0 | 1 | 1 | 1 | 1 | 0 | 0 | 0 | 1 | 0 |
| EC004 | CM | 1 | 0 | 0 | 1 | 1 | 0 | 1 | 0 | 0 | 0 | 1 | 0 |
| EC005 | CM | 0 | 0 | 1 | 0 | 1 | 0 | 0 | 1 | 0 | 0 | 1 | 1 |
| EC006 | CM | 1 | 0 | 0 | 0 | 0 | 1 | 0 | 0 | 0 | 0 | 1 | 0 |
| EC007 | CM | 1 | 1 | 0 | 1 | 1 | 1 | 1 | 0 | 0 | 0 | 0 | 0 |
| EC008 | CM | 1 | 1 | 1 | 1 | 1 | 0 | 1 | 0 | 0 | 1 | 0 | 0 |
| EC009 | CM | 1 | 1 | 0 | 0 | 1 | 0 | 0 | 0 | 0 | 0 | 0 | 0 |
| EC010 | CM | 1 | 0 | 1 | 0 | 1 | 1 | 1 | 0 | 0 | 0 | 0 | 0 |
| EC011 | CM | 1 | 0 | 1 | 0 | 1 | 1 | 0 | 0 | 0 | 0 | 1 | 0 |
| EC012 | CM | 0 | 0 | 0 | 0 | 1 | 0 | 1 | 0 | 1 | 0 | 0 | 0 |
| EC013 | CM | 0 | 1 | 0 | 1 | 0 | 1 | 1 | 0 | 0 | 0 | 0 | 0 |
| EC014 | CM | 1 | 1 | 1 | 0 | 0 | 0 | 1 | 0 | 1 | 0 | 1 | 0 |
| EC015 | CM | 1 | 0 | 1 | 1 | 1 | 1 | 0 | 0 | 0 | 0 | 1 | 0 |
| EC016 | CM | 0 | 0 | 1 | 1 | 1 | 0 | 0 | 0 | 0 | 0 | 1 | 0 |
| EC017 | CM | 1 | 1 | 1 | 1 | 1 | 0 | 1 | 0 | 0 | 0 | 1 | 0 |
| EC018 | CM | 0 | 0 | 1 | 1 | 0 | 0 | 0 | 0 | 0 | 0 | 0 | 0 |
| EC019 | CM | 0 | 1 | 0 | 0 | 1 | 0 | 0 | 0 | 0 | 0 | 0 | 0 |
| EC020 | CM | 1 | 1 | 0 | 0 | 1 | 0 | 1 | 0 | 0 | 1 | 0 | 0 |
| EC021 | CM | 0 | 1 | 0 | 0 | 1 | 1 | 0 | 0 | 0 | 0 | 0 | 0 |
| EC022 | CM | 1 | 0 | 1 | 0 | 0 | 0 | 0 | 0 | 1 | 1 | 0 | 0 |
| EC023 | CM | 0 | 0 | 1 | 1 | 1 | 1 | 1 | 0 | 0 | 1 | 1 | 0 |
| EC024 | CM | 1 | 0 | 1 | 1 | 0 | 0 | 1 | 0 | 0 | 0 | 1 | 0 |
| EC025 | CM | 1 | 1 | 1 | 1 | 1 | 0 | 0 | 0 | 1 | 0 | 0 | 0 |
| EC026 | CM | 1 | 0 | 0 | 1 | 1 | 1 | 1 | 0 | 0 | 0 | 1 | 0 |
| EC027 | CM | 1 | 0 | 1 | 0 | 0 | 0 | 1 | 0 | 0 | 1 | 0 | 0 |
| EC028 | CM | 1 | 0 | 0 | 0 | 0 | 1 | 0 | 0 | 1 | 0 | 0 | 0 |
| EC029 | CM | 0 | 0 | 0 | 0 | 1 | 1 | 1 | 0 | 1 | 0 | 1 | 0 |
| EC030 | CM | 1 | 0 | 0 | 0 | 1 | 0 | 1 | 0 | 0 | 0 | 0 | 0 |
| EC031 | CM | 0 | 1 | 0 | 0 | 1 | 1 | 1 | 0 | 1 | 0 | 1 | 0 |
| EC032 | CM | 0 | 0 | 1 | 0 | 1 | 0 | 1 | 0 | 0 | 0 | 1 | 0 |
| EC033 | CM | 1 | 0 | 0 | 0 | 1 | 1 | 0 | 0 | 0 | 0 | 1 | 0 |
| EC034 | CM | 0 | 0 | 1 | 0 | 1 | 1 | 1 | 0 | 0 | 0 | 1 | 0 |
| EC035 | CM | 1 | 0 | 1 | 0 | 1 | 0 | 1 | 0 | 0 | 0 | 0 | 0 |
| EC036 | CM | 0 | 1 | 1 | 1 | 0 | 1 | 0 | 0 | 0 | 0 | 0 | 0 |
| EC037 | CM | 0 | 0 | 0 | 0 | 1 | 1 | 0 | 0 | 0 | 0 | 0 | 0 |
| EC038 | CM | 0 | 1 | 1 | 0 | 0 | 0 | 0 | 0 | 0 | 0 | 1 | 0 |
| EC039 | CM | 1 | 0 | 0 | 0 | 1 | 1 | 0 | 0 | 1 | 0 | 1 | 0 |
| EC040 | CM | 1 | 0 | 1 | 0 | 1 | 1 | 1 | 1 | 0 | 0 | 0 | 0 |
| EC041 | CM | 1 | 1 | 1 | 0 | 0 | 1 | 0 | 0 | 0 | 0 | 1 | 0 |
| EC042 | CM | 1 | 0 | 0 | 1 | 0 | 0 | 1 | 0 | 0 | 1 | 0 | 0 |
| EC043 | CM | 1 | 0 | 1 | 1 | 1 | 0 | 1 | 0 | 1 | 0 | 0 | 0 |
| EC044 | CM | 1 | 1 | 0 | 1 | 1 | 1 | 0 | 0 | 1 | 1 | 1 | 0 |
| EC045 | CM | 0 | 1 | 0 | 1 | 0 | 0 | 1 | 0 | 1 | 1 | 1 | 0 |
| EC046 | CM | 0 | 0 | 0 | 0 | 0 | 1 | 0 | 0 | 0 | 1 | 0 | 0 |
| EC047 | CM | 1 | 0 | 0 | 0 | 0 | 1 | 1 | 0 | 0 | 0 | 0 | 0 |
| EC048 | CM | 1 | 0 | 1 | 0 | 0 | 1 | 1 | 0 | 0 | 0 | 1 | 0 |
| EC049 | CM | 0 | 0 | 0 | 0 | 1 | 1 | 0 | 0 | 0 | 0 | 0 | 0 |
| EC050 | CM | 0 | 0 | 1 | 1 | 1 | 0 | 0 | 0 | 0 | 0 | 0 | 0 |
| EC051 | CM | 0 | 0 | 1 | 0 | 1 | 1 | 1 | 0 | 0 | 0 | 0 | 0 |
| EC052 | CM | 1 | 0 | 1 | 0 | 1 | 0 | 0 | 0 | 0 | 1 | 1 | 0 |
| EC053 | CM | 1 | 1 | 1 | 1 | 0 | 1 | 1 | 1 | 0 | 0 | 1 | 0 |
| EC054 | CM | 1 | 1 | 0 | 0 | 1 | 0 | 1 | 0 | 0 | 1 | 0 | 0 |
| EC055 | CM | 0 | 1 | 0 | 1 | 1 | 1 | 1 | 0 | 1 | 1 | 0 | 1 |
| EC056 | CM | 0 | 1 | 0 | 0 | 1 | 1 | 0 | 0 | 1 | 0 | 0 | 0 |
| EC057 | CM | 1 | 0 | 1 | 0 | 1 | 1 | 0 | 0 | 1 | 1 | 1 | 1 |
| EC058 | CM | 1 | 0 | 0 | 1 | 0 | 0 | 1 | 0 | 0 | 0 | 1 | 0 |
| EC059 | CM | 1 | 1 | 1 | 0 | 1 | 1 | 1 | 0 | 0 | 0 | 1 | 0 |
| EC060 | CM | 0 | 1 | 1 | 1 | 0 | 0 | 1 | 1 | 1 | 0 | 1 | 0 |
| EC061 | CM | 0 | 0 | 1 | 0 | 0 | 1 | 0 | 0 | 0 | 0 | 1 | 0 |
| EC062 | CM | 1 | 1 | 1 | 0 | 1 | 1 | 0 | 0 | 0 | 0 | 0 | 0 |
| EC063 | CM | 1 | 1 | 1 | 1 | 1 | 0 | 0 | 0 | 0 | 1 | 0 | 0 |
| EC064 | CM | 0 | 0 | 0 | 1 | 0 | 1 | 0 | 0 | 0 | 1 | 1 | 0 |
| EC065 | CM | 1 | 1 | 1 | 1 | 0 | 1 | 1 | 0 | 1 | 0 | 1 | 0 |
| EC066 | CM | 1 | 0 | 1 | 1 | 0 | 0 | 0 | 1 | 1 | 0 | 0 | 0 |
| EC067 | CM | 1 | 0 | 1 | 0 | 1 | 0 | 1 | 0 | 0 | 1 | 0 | 0 |
| EC068 | CM | 0 | 0 | 1 | 1 | 0 | 1 | 1 | 0 | 1 | 0 | 1 | 0 |
| EC069 | CM | 0 | 0 | 0 | 0 | 1 | 0 | 0 | 0 | 0 | 0 | 0 | 0 |
| EC070 | CM | 1 | 0 | 1 | 1 | 1 | 1 | 0 | 0 | 0 | 0 | 1 | 1 |
| EC071 | CM | 1 | 1 | 0 | 0 | 0 | 1 | 0 | 0 | 1 | 0 | 1 | 0 |
| EC072 | CM | 0 | 0 | 1 | 1 | 0 | 1 | 0 | 0 | 1 | 0 | 0 | 0 |
| EC073 | CM | 0 | 0 | 1 | 0 | 0 | 1 | 1 | 0 | 0 | 0 | 1 | 0 |
| EC074 | CM | 1 | 0 | 1 | 0 | 1 | 0 | 0 | 0 | 0 | 0 | 0 | 0 |
| EC075 | CM | 1 | 1 | 1 | 1 | 1 | 1 | 1 | 0 | 0 | 0 | 0 | 0 |
| EC076 | CM | 0 | 1 | 1 | 0 | 0 | 1 | 1 | 0 | 0 | 0 | 0 | 0 |
| EC077 | CM | 1 | 0 | 0 | 1 | 1 | 1 | 1 | 0 | 0 | 0 | 0 | 0 |
| EC078 | CM | 0 | 1 | 0 | 0 | 1 | 0 | 0 | 0 | 0 | 0 | 1 | 0 |
| EC079 | CM | 0 | 0 | 1 | 0 | 0 | 1 | 0 | 0 | 0 | 0 | 0 | 0 |
| EC080 | CM | 1 | 1 | 0 | 1 | 1 | 1 | 0 | 0 | 1 | 1 | 1 | 0 |
| EC081 | CM | 0 | 1 | 1 | 0 | 1 | 0 | 1 | 0 | 1 | 0 | 0 | 0 |
| EC082 | CM | 0 | 1 | 0 | 0 | 1 | 0 | 0 | 0 | 0 | 0 | 0 | 0 |
| EC083 | CM | 1 | 0 | 1 | 1 | 0 | 0 | 0 | 0 | 0 | 0 | 0 | 0 |
| EC084 | CM | 1 | 0 | 0 | 1 | 0 | 1 | 1 | 0 | 0 | 0 | 0 | 0 |
| EC085 | CM | 1 | 1 | 1 | 0 | 1 | 0 | 0 | 0 | 0 | 0 | 1 | 0 |
| EC086 | CM | 1 | 0 | 1 | 0 | 0 | 1 | 1 | 0 | 0 | 1 | 1 | 0 |
| EC087 | CM | 0 | 1 | 1 | 0 | 1 | 1 | 0 | 0 | 0 | 0 | 1 | 0 |
| EC088 | CM | 1 | 0 | 1 | 0 | 0 | 0 | 1 | 0 | 0 | 0 | 1 | 1 |
| EC089 | CM | 1 | 0 | 1 | 1 | 1 | 0 | 1 | 0 | 0 | 1 | 0 | 0 |
| EC090 | CM | 1 | 1 | 0 | 0 | 0 | 0 | 0 | 0 | 0 | 0 | 0 | 0 |
| EC091 | CM | 0 | 1 | 0 | 0 | 1 | 1 | 1 | 0 | 0 | 0 | 1 | 0 |
| EC092 | CM | 0 | 0 | 1 | 1 | 1 | 0 | 1 | 0 | 1 | 1 | 1 | 0 |
| EC093 | CM | 1 | 1 | 1 | 0 | 0 | 0 | 1 | 0 | 1 | 0 | 1 | 0 |
| EC094 | CM | 0 | 0 | 1 | 1 | 1 | 0 | 0 | 0 | 0 | 0 | 0 | 0 |
| EC095 | CM | 0 | 1 | 1 | 1 | 1 | 1 | 1 | 0 | 0 | 0 | 0 | 0 |
| EC096 | SM | 1 | 0 | 1 | 0 | 0 | 1 | 1 | 0 | 0 | 0 | 0 | 0 |
| EC097 | SM | 0 | 1 | 0 | 1 | 1 | 1 | 1 | 0 | 0 | 0 | 0 | 0 |
| EC098 | SM | 1 | 1 | 0 | 1 | 1 | 1 | 1 | 0 | 0 | 0 | 0 | 0 |
| EC099 | SM | 1 | 1 | 0 | 0 | 1 | 1 | 1 | 0 | 0 | 0 | 1 | 0 |
| EC100 | SM | 0 | 1 | 1 | 0 | 1 | 1 | 1 | 0 | 1 | 0 | 1 | 0 |
| EC101 | SM | 1 | 1 | 0 | 1 | 1 | 1 | 0 | 0 | 0 | 0 | 1 | 0 |
| EC102 | SM | 1 | 1 | 0 | 0 | 1 | 0 | 0 | 0 | 0 | 0 | 1 | 0 |
| EC103 | SM | 0 | 0 | 1 | 0 | 1 | 1 | 1 | 0 | 0 | 0 | 1 | 0 |
| EC104 | SM | 0 | 0 | 1 | 0 | 1 | 1 | 0 | 0 | 0 | 0 | 1 | 0 |
| EC105 | SM | 0 | 1 | 1 | 0 | 0 | 0 | 0 | 0 | 0 | 0 | 0 | 0 |
| EC106 | SM | 1 | 0 | 0 | 0 | 1 | 0 | 1 | 0 | 1 | 0 | 0 | 0 |
| EC107 | SM | 0 | 1 | 0 | 1 | 1 | 0 | 1 | 0 | 0 | 0 | 0 | 1 |
| EC108 | SM | 0 | 1 | 0 | 0 | 1 | 1 | 1 | 0 | 0 | 0 | 0 | 0 |
| EC109 | SM | 0 | 0 | 1 | 0 | 1 | 1 | 1 | 0 | 0 | 0 | 1 | 0 |
| EC110 | SM | 1 | 1 | 0 | 1 | 0 | 0 | 1 | 0 | 1 | 0 | 1 | 0 |
| EC111 | SM | 0 | 1 | 0 | 1 | 1 | 1 | 1 | 0 | 0 | 0 | 0 | 0 |
| EC112 | SM | 1 | 0 | 1 | 0 | 0 | 0 | 1 | 0 | 0 | 0 | 0 | 0 |
| EC113 | SM | 0 | 1 | 0 | 0 | 1 | 1 | 1 | 0 | 0 | 0 | 0 | 1 |
| EC114 | SM | 0 | 1 | 1 | 0 | 1 | 0 | 1 | 0 | 1 | 0 | 0 | 0 |
| EC115 | SM | 1 | 0 | 0 | 1 | 1 | 1 | 0 | 0 | 0 | 0 | 1 | 0 |
| EC116 | SM | 1 | 1 | 1 | 0 | 0 | 1 | 1 | 0 | 1 | 0 | 0 | 0 |
| EC117 | SM | 1 | 0 | 1 | 1 | 1 | 1 | 1 | 0 | 0 | 0 | 0 | 1 |
| EC118 | SM | 0 | 0 | 0 | 0 | 1 | 0 | 1 | 0 | 1 | 0 | 1 | 0 |
| EC119 | SM | 0 | 1 | 1 | 0 | 1 | 1 | 1 | 0 | 0 | 0 | 0 | 0 |
| EC120 | SM | 1 | 1 | 1 | 0 | 1 | 0 | 1 | 0 | 0 | 0 | 1 | 1 |
| EC121 | SM | 0 | 1 | 1 | 0 | 0 | 0 | 1 | 0 | 1 | 0 | 0 | 0 |
| EC122 | SM | 1 | 1 | 0 | 0 | 1 | 0 | 1 | 0 | 0 | 0 | 1 | 0 |
| EC123 | SM | 0 | 1 | 1 | 1 | 0 | 0 | 0 | 0 | 0 | 0 | 1 | 0 |
| EC124 | SM | 0 | 0 | 0 | 0 | 1 | 0 | 1 | 0 | 0 | 0 | 1 | 0 |
| EC125 | SM | 1 | 0 | 1 | 1 | 1 | 0 | 1 | 0 | 0 | 0 | 1 | 0 |
| EC126 | SM | 0 | 0 | 1 | 0 | 1 | 1 | 1 | 0 | 0 | 0 | 0 | 0 |
| EC127 | SM | 1 | 0 | 0 | 1 | 1 | 1 | 0 | 0 | 0 | 0 | 1 | 0 |
| EC128 | SM | 0 | 0 | 0 | 1 | 0 | 0 | 1 | 0 | 0 | 0 | 1 | 0 |
| EC129 | SM | 1 | 1 | 1 | 1 | 1 | 0 | 1 | 0 | 1 | 0 | 1 | 1 |
| EC130 | SM | 1 | 1 | 0 | 0 | 1 | 0 | 1 | 0 | 1 | 0 | 0 | 0 |
| EC131 | SM | 1 | 0 | 0 | 1 | 1 | 1 | 1 | 0 | 1 | 0 | 1 | 0 |
| EC132 | SM | 0 | 1 | 1 | 0 | 0 | 1 | 1 | 0 | 0 | 0 | 0 | 0 |
| EC133 | SM | 0 | 1 | 1 | 0 | 0 | 0 | 0 | 0 | 0 | 0 | 0 | 0 |
| EC134 | SM | 1 | 1 | 1 | 0 | 1 | 0 | 0 | 0 | 0 | 0 | 0 | 0 |
| EC135 | SM | 1 | 1 | 0 | 1 | 1 | 0 | 0 | 0 | 0 | 0 | 1 | 0 |

**Supplementary Table S8**. Phylogenetic distribution of ESBL-producing *Escherichia coli* isolates from clinical (CM) and subclinical (SM) mastitis.

| **Phylogroup** | **CM (n = 95)** | **SM (n = 40)** | **OR (95% CI)** | **p-value** |
| --- | --- | --- | --- | --- |
| **A** | 15 (15.8%) | 10 (25.0%) | 0.56 (0.22–1.44) | 0.22 |
| **B1** | 5 (5.3%) | 5 (12.5%) | 0.39 (0.10–1.57) | 0.18 |
| **B2** | 45 (47.4%) | 10 (25.0%) | **2.82 (1.06–7.04)** | **0.04** |
| **C** | 5 (5.3%) | 5 (12.5%) | 0.39 (0.10–1.57) | 0.18 |
| **D** | 10 (10.5%) | 5 (12.5%) | 0.82 (0.26–2.64) | 0.75 |
| **E** | 15 (15.8%) | 5 (12.5%) | 1.32 (0.43–4.03) | 0.63 |
| **F** | 0 (0%) | 10 (25.0%) | — | 0.281 |

**Supplementary Table S9.** Statistical comparison of phenotypic and molecular characteristics of *Escherichia coli* isolates between clinical (CM) and subclinical (SM) mastitis cases.

| **Category** |  | **Chi²** | **p (Chi²)** | **p (Fisher)** | **OR** | **CI-Low** | **CI-High** | **Cramer V** |
| --- | --- | --- | --- | --- | --- | --- | --- | --- |
| **Antibiotic** | **AMC** | 1.776 | 0.183 | 0.231 | 1.75 | 0.76 | 4.01 | 0.115 |
|  | **FOX** | — | — | 1 | — | — | — | — |
|  | **CTX** | — | — | 1 | — | — | — | — |
|  | **CRO** | — | — | 1 | — | — | — | — |
|  | **CAZ** | **35.26** | **2.9×10⁻⁹** | **1.36×10⁻⁹** | **15.17** | 5.4 | 42.57 | **0.511** |
|  | **ATM** | **42.68** | **6.46×10⁻¹¹** | **3.57×10⁻¹¹** | **19.6** | 6.91 | 55.59 | **0.562** |
|  | **IMP** | — | — | 1 | — | — | — | — |
|  | **CIP** | **37.33** | **9.95×10⁻¹⁰** | **7.71×10⁻⁹** | **18** | 6.03 | 53.71 | **0.526** |
|  | **LEV** | **42.68** | **6.46×10⁻¹¹** | **3.57×10⁻¹¹** | **19.6** | 6.91 | 55.59 | **0.562** |
|  | **STX** | **29.46** | **5.72×10⁻⁸** | **1.87×10⁻⁷** | **8.89** | 3.82 | 20.69 | **0.467** |
|  | **C** | **19.89** | **8.18×10⁻⁶** | **6.24×10⁻⁷** | — | — | — | 0.384 |
|  | **CN** | **7.11** | **0.0077** | **0.0055** | — | — | — | 0.229 |
| **Resistance gene  (Solitary Vs Combinations)** | **CTX-M alone** | 2.964 | 0.085 | 0.105 | 0.47 | 0.2 | 1.12 | 0.148 |
|  | **TEM alone** | 4.821 | **0.028** | 0.086 | — | — | — | 0.189 |
|  | **SHV alone** | 2.297 | 0.13 | 0.154 | 0.27 | 0.04 | 1.65 | 0.13 |
|  | **CTX-M + TEM** | 0.282 | 0.595 | 0.66 | 1.28 | 0.52 | 3.16 | 0.046 |
|  | **CTX-M + SHV** | 0.854 | 0.355 | 0.408 | 0.62 | 0.22 | 1.73 | 0.08 |
|  | **TEM + SHV** | 0.82 | 0.365 | 0.582 | 0.41 | 0.06 | 3.01 | 0.078 |
|  | **TRIPLE (CTX-M + TEM + SHV)** | **15.529** | **0.000081** | **0.00004** | **8.97** | 2.58 | 31.15 | **0.339** |

| **Resistance gene  (Total)** | **blaCTX-M** | **11.1** | **0.00086** | **0.00186** | **6** | 1.9 | 18.96 | **0.287** |
| --- | --- | --- | --- | --- | --- | --- | --- | --- |
|  | **blaTEM** | **11.15** | **0.00084** | **0.00111** | **3.61** | 1.67 | 7.82 | **0.287** |
|  | **blaSHV** | **4.69** | **0.0303** | **0.0383** | **2.29** | 1.07 | 4.89 | **0.186** |
| **Virulence gene** | **iutA** | 0.711 | 0.399 | 0.45 | 1.38 | 0.65 | 2.89 | 0.073 |
|  | **iroN** | **4.69** | **0.03** | **0.038** | **0.44** | 0.2 | 0.93 | **0.186** |
|  | **iucD** | 0.711 | 0.399 | 0.45 | 1.38 | 0.65 | 2.89 | 0.073 |
|  | **iss** | 0.247 | 0.619 | 0.703 | 1.21 | 0.57 | 2.59 | 0.043 |
|  | **ompT** | 1.776 | 0.183 | 0.231 | 0.57 | 0.25 | 1.31 | 0.115 |
|  | **hlyA** | 0.078 | 0.78 | 0.851 | 1.11 | 0.53 | 2.33 | 0.024 |
|  | **hlyF** | **5.834** | **0.0157** | **0.021** | **0.37** | 0.16 | 0.84 | **0.208** |
|  | **tsh** | 2.186 | 0.139 | 0.321 | — | — | — | 0.127 |
|  | **vat** | 0.025 | 0.873 | 1 | 1.07 | 0.46 | 2.5 | 0.014 |
|  | **colV** | **9.886** | **0.0017** | **0.00086** | — | — | — | **0.271** |
|  | **papC** | 0.078 | 0.78 | 0.851 | 0.9 | 0.43 | 1.88 | 0.024 |
|  | **papG2A** | 2.149 | 0.143 | 0.161 | 0.39 | 0.11 | 1.43 | 0.126 |
| **Integron** | **intI1 alone** | 1.776 | 0.183 | 0.231 | 0.57 | 0.25 | 1.31 | 0.115 |
|  | **intI2 alone** | — | — | 1 | — | — | — | — |
|  | **Both intI1 + intI2** | **4.547** | **0.033** | **0.0332** | — | — | — | **0.184** |
|  | **None** | 0.025 | 0.873 | 1 | 1.07 | 0.46 | 2.5 | 0.014 |
| **Phylotype** | **A** | 1.491 | 0.222 | 0.223 | 0.56 | 0.22 | 1.44 | 0.101 |
|  | **B1** | 1.786 | 0.181 | 0.188 | 0.39 | 0.1 | 1.57 | 0.122 |
|  | **B2** | **4.26** | **0.039** | **0.044** | **2.82** | **1.06** | 7.04 | **0.189** |
|  | **C** | 1.786 | 0.181 | 0.188 | 0.39 | 0.1 | 1.57 | 0.122 |
|  | **D** | 0.096 | 0.756 | 0.766 | 0.82 | 0.26 | 2.64 | 0.024 |
|  | **E** | 0.235 | 0.627 | 0.76 | 1.32 | 0.43 | 4.03 | 0.039 |
|  | **F** | **8.99** | **0.0027** | **0.0009** | — | — | — | **0.281** |


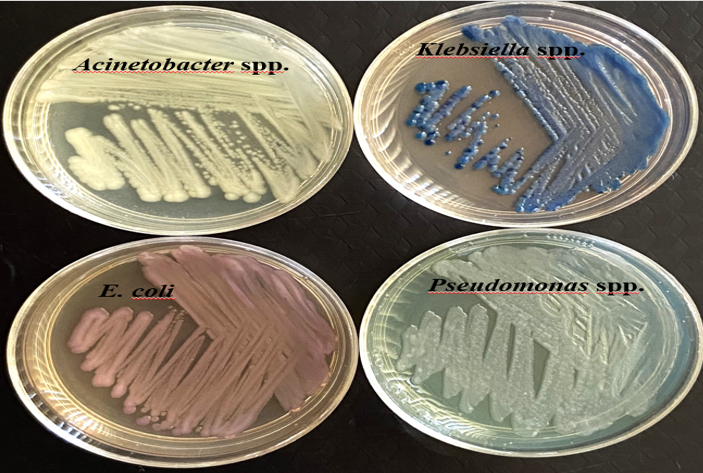


**Supplementary Figure S1:** Typical colony morphologies of *Acinetobacter*, *Klebsiella*, *E.coli*, and *Pseudomonas* spp. on CHROMagar™ Orientation, demonstrating the characteristic pigmentation patterns used for preliminary species differentiation.


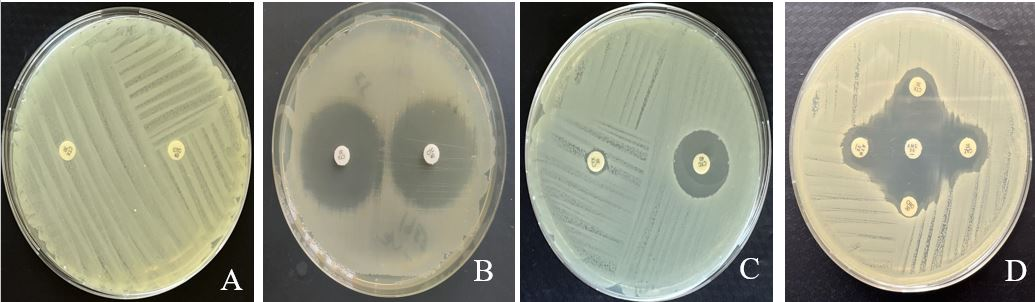


**Supplementary Figure S2:** Phenotypic confirmation of ESBL production using (A–C) Combined Disk Test (CDT) and (D) Double Disk Synergy Test (DDST). Enhancement of inhibition zones around clavulanic acid–containing disks indicates ESBL positivity.


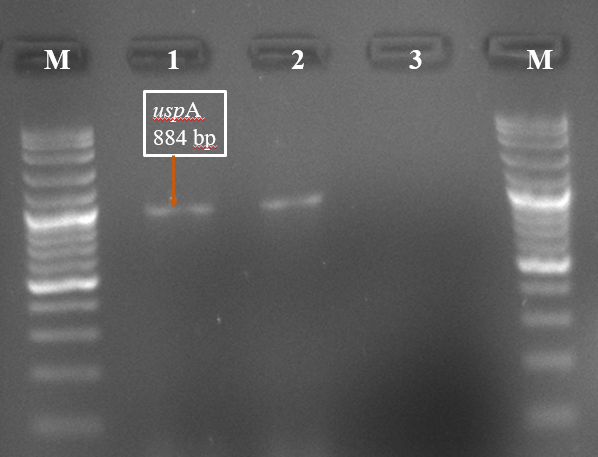


**Supplementary Figure S3:** Molecular identification of *E. coli* 1. *uspA* gene (884 bp) *E. coli* ATCC 35150 (Positive control) 2. *E*. *coli* field isolate 3. *S*. Typhimurium ATCC 14028 (Negative Control).


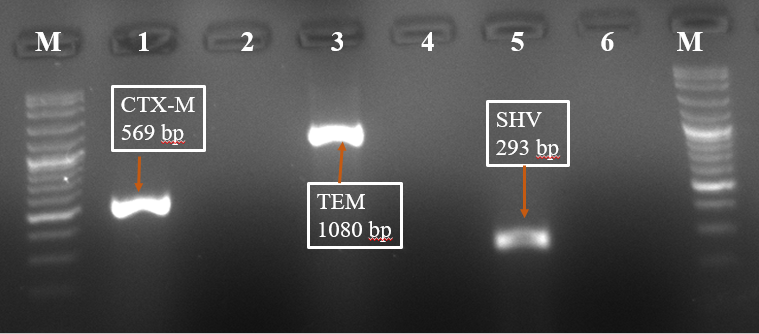


**Supplementary Figure S4:** Gel electrophoresis image of PCR amplification products of ESBL genes. 1. *blaCTX-*M (569 bp), 3. *blaTEM* (1080 bp) 5. *blaSHV* (293 bp), 2,4,6: Negative control (master mix without DNA).


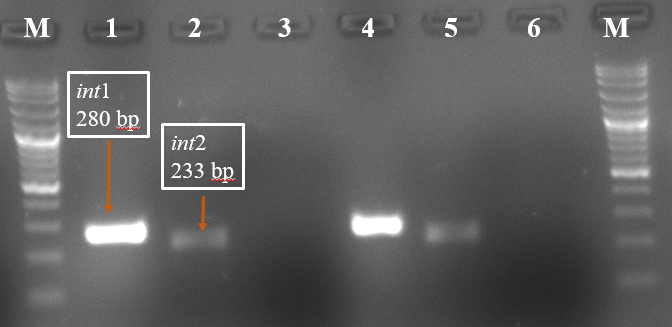


**Supplementary Figure S5:** Gel electrophoresis image of PCR amplification products of integron genes. 1. *int1* gene (280 bp) Sequenced field strain (Positive Control), 2. *int2* gene (233 bp) Sequenced field strain (Positive Control), 2. Field strain (*int1* gene positive), 5. Field strain (*int2* gene positive), 3,6. Negative control (DNA-free master mix).


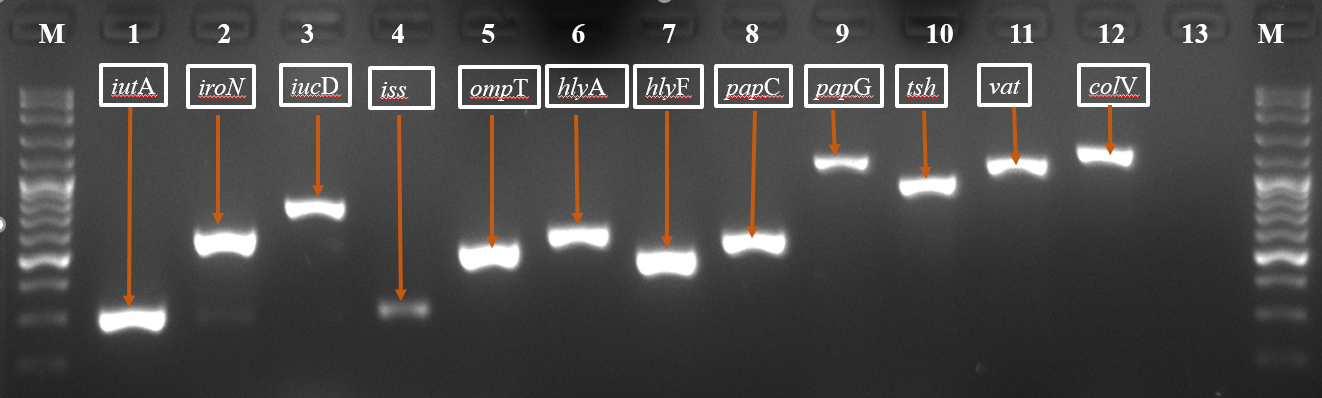


**Supplementary Figure S6:** Gel electrophoresis image of virulence genes of *E. coli* strains isolated from cows with mastitis. 1. *iutA* (302 bp), 2. *iroN* (553 bp), 3. *iucD* (714 bp), 4. *iss* (290 bp), 5. *ompT* (496 bp), 6. *hlyA* (534 bp), 7. *hlyF* (450 bp), 8. *papC* (500 bp), 9. *papG2A* (1070 bp), 10. *tsh* (825 bp), 11. *vat* (981 bp), 12. *colV* (1180 bp) genes positive isolates; 13. Negative control (master mix without DNA). M. 100 bp DNA ladder (Vivantis).


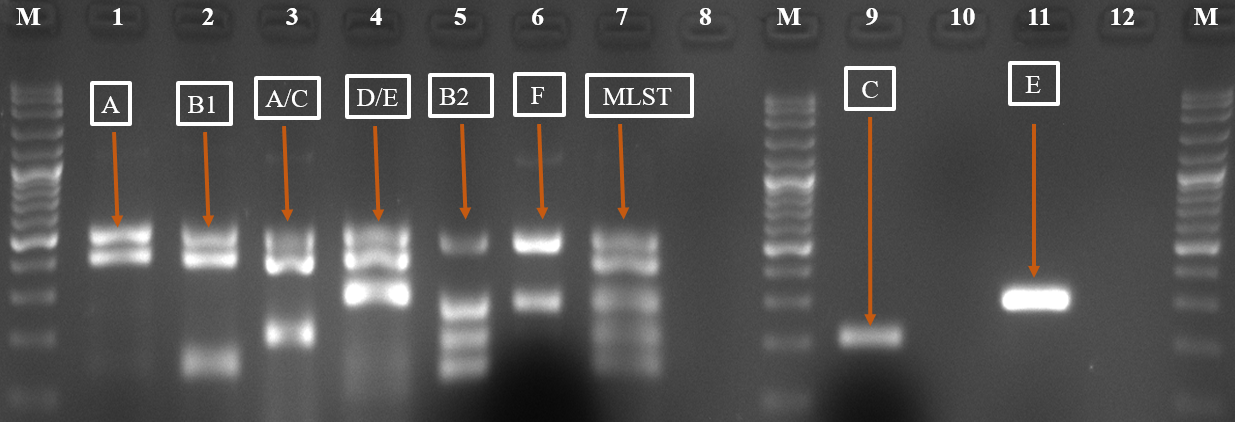
**Supplementary Figure S7:** Phylotype profiles of *E. coli* isolates according to the new Clermont phylotyping method. M: Marker (100 bp, Vivantis), 1. Group A (400 bp, 489 bp), 2. Group B1 (152 bp, 400 bp, 489 bp), 3. Group A/C (211 bp, 400 bp, 489 bp), 4. Group D/E (288 bp, 400 bp, 489 bp), 5. Group B2 (152 bp, 211 bp, 288 bp, 489 bp), 6. Group (288 bp, bp), 7. Unknown Group (?) (Obtained from previous studies) (++ + + + +) (152 bp, 211 bp, 288 bp, 400 bp, 489 bp), 8: S. Enteritidis ATCC 13076 (Negative Control) 9. Group C (219 bp) 11. Group E (301 bp), 10, 12: DNA-free master mix (Negative Control), M: Marker (100 bp, Fermentas).
